# Volumetric printed biomimetic scaffolds support in vitro lactation of human milk-derived mammary epithelial cells

**DOI:** 10.1126/sciadv.adu5793

**Published:** 2025-06-04

**Authors:** Amelia Hasenauer, Kajetana Bevc, Maxwell C. McCabe, Parth Chansoria, Anthony J. Saviola, Kirk C. Hansen, Karen L. Christman, Marcy Zenobi-Wong

**Affiliations:** ^1^ Tissue Engineering + Biofabrication Laboratory, Department of Health Sciences and Technology, ETH Zürich, Otto-Stern-Weg 7, 8093 Zürich, Switzerland.; ^2^ Department of Biochemistry and Molecular Genetics, School of Medicine, University of Colorado, 12801 E 17th Ave., Aurora, CO 80045, USA.; ^3^ Shu Chien-Gene Lay Department of Bioengineering, Sanford Stem Cell Institute, Sanford Consortium for Regenerative Medicine, UC San Diego, San Diego, CA 92093, USA.

## Abstract

The human breast is remarkably plastic and remodels with each birth to produce milk optimally suited for the changing demands of the newborn. This dynamic nature of lactation makes it challenging to study under controlled conditions. Given the health benefits of human milk, models of secretory mammary tissue would offer opportunities to study factors that influence this important food source. First, 3D models of the mammary duct/alveoli (D/A) were designed inspired by shapes found in vivo. Photoresins based on mammary decellularized extracellular matrix (dECM) were optimized to match the mechanical properties of native breast tissue. Next, these D/A models were printed with a volumetric printer and seeded with human milk-derived mammary epithelial cells (MECs). MECs formed stable epithelial layers on the printed surfaces and secreted the β-casein and milk fat globules. This model offers exciting avenues to explore hormonal, nutritional, and mechanobiological factors involved in lactation, thereby improving understanding of lactation for the benefit of infants and their mothers.

## Teaser

3D-printed breast tissue models pave the way for cutting-edge lactation research.

## INTRODUCTION

Human breast milk is uniquely adapted to meet the infant’s nutritional needs, supports immune function, shapes the gut microbiome, and protects against multiple debilitating childhood diseases (*1*–*4*). Despite the importance of newborn nutrition for early and long-term infant health, our understanding of human milk and lactation biology remains incomplete. Milk production is influenced by various factors such as maternal diet, hormones, and medications, but research involving breastfeeding mothers is often limited to correlative studies, which can yield inconclusive results (*5*). Three-dimensional (3D)–printed in vitro lactation models hold the potential to study milk secretion under controlled conditions and provide fundamental insights into lactation biology to support mothers across all socioeconomic strata (*4*, *6*, *7*).

A subset of mammary epithelial cells (MECs) is the milk-producing cells of the mammary gland. Luminal MECs, i.e., lactocytes, synthesize milk components supported by basal MECs that contract to facilitate milk expression (*8*–*10*). Bissell and colleagues (*11*, *12*) have fundamentally reshaped our understanding of the extracellular matrix (ECM) and emphasize its role beyond a mere passive support scaffold to an active regulator of cell signaling and behavior. MECs interact with the basement membrane (BM) and underlying ECM through integrin-mediated binding and respond to mechanical and biochemical stimuli of the ECM, to induce cell polarization along the basal/apical axis, tight junction formation, and tissue self-reorganization (*5*–*7**,* *13*).

**Fig. 1.**
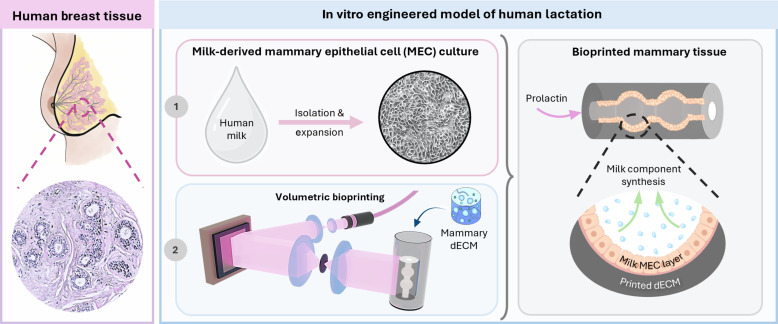
3D printing of human alveolar tissue. **Left**: In human breast tissue, alveoli are the milk-producing units during lactation. **Right**: (1) Milk-derived MECs and (2) volumetrically printed (VP) dECM are leveraged to create secretory in vitro models. Expanded milk MECs are postseeded in the printed mammary tissue structures where they synthesize milk.

Despite recent advances in tissue engineering, there are very few models of in vitro lactation, and those that exist rely heavily on organoids. Organoid technology is a versatile tool that allows one to explore the mammary gland in vitro from development to breast cancer. However, organoids face limitations such as low formation rates, limited life span, and variations in shape and/or size that contribute to experimental variability (*14*, *15*). Furthermore, their use precludes the continuous collection and analysis of secreted milk proteins without cell layer disruption. Most in vitro models rely on murine cells, which vary in developmental timing, cell diversity, and ECM composition compared to humans (*16*, *17*). In addition, murine milk differs from human milk in cell, protein, fat, and carbohydrate content, reflecting species-specific nutritional needs (*18*, *19*). These differences can compromise the translational relevance of organoid work, which further underlines the need for alternative human breast models. To propose a fresh approach, we used light-based 3D printing to biofabricate human lactation models composed of decellularized ECM (dECM) from bovine mammary tissue and human milk-derived MECs (Fig. 1).

To date, the literature reports only a few studies that involve bioprinting of MECs, primarily focused on breast cancer organoids combined with extrusion bioprinting (*20*, *21*). In these studies, the placement and number of cells (or organoids) within the biomaterial can be precisely controlled, which reduces experimental variability (*15*, *22*). However, current extrusion-based approaches have not successfully replicated the architecture of mammary ducts and alveoli, primarily due to challenges associated with printing overhanging hollow shapes. Thus, current studies rely on the cells’ ability to self-organize into these structures, which restricts the spatial control over the epithelial and stromal compartment’s architecture, particularly given the inherent biological variability in tissue formation (*21*).

In contrast, recent advancements in volumetric printing (VP) overcome the limitations of conventional layer-by-layer additive manufacturing and allow for the printing of perfusable tissue structures. In VP, the 3D model is discretized into a series of images that are projected onto a rotating vat filled with photoresin. The voxels, where the accumulation of photons surpasses the gelation threshold, undergo solidification, while the remaining resin can be washed away. This state-of-the-art technology marries high resolution with fast printing speeds (several seconds) to produce defect-free structures with precisely controllable surface topographies, which makes it an attractive yet unexploited choice for engineering mammary tissue (*23*, *24*).

The recent progress in hardware printing has been complemented by advances in biomaterials. One such innovation is tissue decellularization, which transforms organs and tissues into cutting-edge biomaterials that can be directly light printed using VP without any chemical modification (*25*, *26*). The interest in dECM materials stems from the preservation of soluble proteins during decellularization, which enhances the extracted biomaterials’ bioactivity and reduces cytotoxicity. This makes dECM-based resins more conducive to cell-matrix interactions, proliferation, and differentiation compared to single component resins such as methacrylated biomaterials (e.g., Gelatin Methacryloyl) and ultimately facilitates the development of physiologically relevant engineered tissues (*27*–*29*).

To recreate tissue models in vitro, MECs have traditionally been isolated from mammoplasties; however, human milk has emerged as a valuable source of these cells. Recent advances in omic technologies have revealed the presence of MECs, predominantly lactocytes, along with a population of mammary epithelial progenitor cells in breast milk (*30*, *31*). Milk MECs can be noninvasively isolated and retain key characteristics of the mammary epithelium, including the ability to form acinar structures in vitro (*32*, *33*). Despite these promising prerequisites, milk MECs have, to date, not been applied in tissue engineering.

In this study, we present a VP lactation model that leverages advanced biomaterials and 3D printing technologies and highlights the suitability of milk MECs for mammary tissue engineering. Our model paves the way for research approaches into human lactation and provides an in vitro platform for exploring the impact of ECM components, as well as the effects of nutrition, hormones, and drugs on milk secretion mechanisms.

## RESULTS

### Milk-derived MECs can be expanded and maintain lactation potential in vitro

**Fig. 2.**
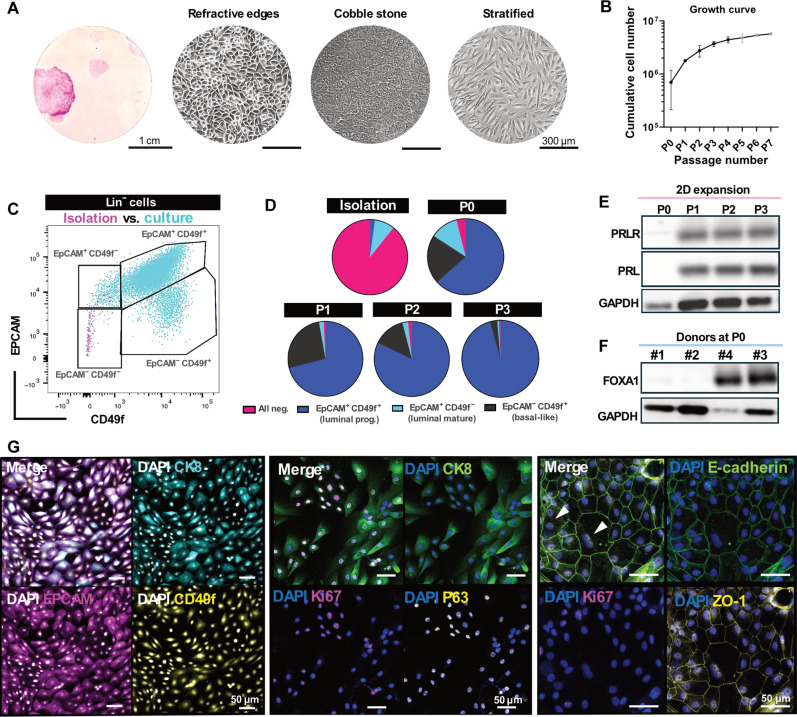
Phenotypic characterization of human milk-derived primary MECs in 2D in vitro culture. (**A**) Milk MECs postisolation (P0) stained with rhodamine B after 12 days of culture (*32*). Milk MECs form colonies of different sizes and cell shapes, as shown with bright-field imaging. (**B**) Milk MECs can be expanded over several passages (*x* axis, number of passages; *y* axis, cumulative cell number); means ± SD, *n* = 4 biological replicates represented in black, *n* = 2 or 1 biological replicate represented in gray. (**C**) Overlay of representative flow cytometry plot showing the cells postisolation in pink [epithelial cell adhesion marker negative (EpCAM^−^) and integrin alpha-6 negative (CD49f^−^)] and during in vitro culture in cyan, demonstrating a shift toward EpCAM^−^/CD49^+^ (basal-like), EpCAM^+^/CD49f^+^ (luminal progenitor), and EpCAM^+^/CD49f^−^ (mature luminal) epithelial cell populations. (**D**) Distribution of MEC populations during isolation and through successive passages (P0 to P3). The color-coded legend denotes the following: pink: all negative (EpCAM^−^ CD49f^−^); dark blue: luminal progenitors (EpCAM^+^ CD49f^+^); light blue: luminal mature cells (EpCAM^+^ CD49f^−^); black: basal-like cells (EpCAM^−^ CD49f^+^). Data are represented as the means of four different donors. (**E**) Western blot showing the presence of prolactin (PRL) and PRLR in the milk MECs over three passages. GAPDH, glyceraldehyde-3-phosphate dehydrogenase. (**F**) Western blot showing the presence of FOXA1-expressing cells in two of the four donors at P0. (**G**) Immunofluorescent staining of milk MECs in 2D in vitro culture on Matrigel-coated polystyrene cell culture flasks with epithelial markers (EpCAM and CD49f), luminal marker (CK8), markers of proliferation (Ki67) and basal myoepithelial cells (P63) (*42*), and markers for tight junction formation (E-cadherin and ZO-1) and proliferation (Ki67). White arrowheads indicate binucleated cells. Scale bars, 50 μm. DAPI, 4′,6-diamidino-2-phenylindole.

Cells derived from human breast milk were expanded to investigate the potential of milk MECs for in vitro lactation models. Human milk contained colony-generating cells with various morphologies, e.g., cobble stone, refractive edges, or stratified, in line with reports on epithelial cells in 2D culture (Fig. 2A and fig. S1) (*34*, *35*). Cells from all donors were expandable over at least three passages with media changes occurring every 2 days or daily after day 4 to accommodate the increased cell number and nutrient consumption (Fig. 2B, figs. S1 and S2, and table S1).

To further characterize the cells postisolation and during expansion, flow cytometric analysis was performed, and epithelial cell adhesion marker (EpCAM) and CD49f (integrin alpha-6) were used as epithelial markers to identify MECs (Fig. 2C and figs. S3 to S8) (*30*). Freshly isolated cells predominantly lacked the expression of EpCAM and/or CD49f (<11% positive for epithelial markers EpCAM and/or CD49f). However, following culture establishment and the first passage, the proportion of EPCAM^+^ and/or CD49f^+^ cells increased to >90%, while CD90 [marking fibroblasts and basal MECs (especially type 1 contractile ductal basal cells)] (*36*) remained below 2% after passage 0 (Fig. 2C and fig. S6). Luminal and basal MECs are crucial for milk synthesis and expression, respectively. Between the donors, the populations of basal-like (CD49f^+^/EPCAM^−^ and CK14^+^) and luminal MECs [CD49f^+^/EPCAM^+^ (progenitors) or CD49f^+^/EPCAM^−^ (mature) and cytokeratin 8 (CK8^+^)] varied substantially between donors and passages (~2 to ~60% basal MECs and ~30 to ~90% luminal MECs between donors), with a trend toward a higher proportion of luminal (predominantly progenitor) cells rather than basal-like cells (Fig. 2D and fig. S4). Together, these results demonstrate the presence of luminal cells (mature and progenitor) and basal-like MECs during 2D culture, with the preservation of epithelial phenotype over passaging (Fig. 2, C and D, and figs. S3 to S8).

The prolactin receptor (PRLR) is crucial for hormonal regulation and the activation of downstream pathways [e.g., signal transducer and activator of transcription 5 (STAT5)] that mediate lactation. Western blot analysis of cultured milk MECs showed the retention of PRLR over several passages, which indicated the suitability for in vitro prolactin stimulation (Fig. 2E and fig. S9). Milk-derived MECs expressed prolactin in a prolactin-free culture medium (Fig. 2E and fig. S9). This could potentially be explained by the cells’ ability to produce prolactin through autocrine signaling (*37*–*39*). The presence of prolactin was not reported in published single-cell RNA sequencing (RNA-seq) studies on human milk cells postisolation (*30*–*32*, *40*). We further observed forkhead box A1 (FOXA1)-positive cells in two of the four donors at P0 during culture by immunofluorescent staining, and the presence of FOXA1-positive cells was reconfirmed by Western blot (Fig. 2F and fig. S10). This suggested that milk-derived MECs can retain hormone-responsive cells in vitro (*41*). The presence of flow cytometry markers (EPCAM and CD49f) was reconfirmed by staining in 2D, and to further determine whether cultured cells were in active cell cycle, MECs were stained with Ki67 for proliferation (Fig. 2F). A fraction of proliferative cells coexpressed the master regulator of basal myoepithelial differentiation, P63 (*42*), and some expressed Alpha-smooth muscle actin (Fig. 2F and figs. S11 and S12). Milk MECs were able to form tight junctions demonstrated by zonulin-1 (ZO-1), E-cadherin, and F-actin colocalization, a key requirement for secretory epithelial tissues to execute their function, which further indicates their potential for mammary tissue engineering (Fig. 2F and fig. S13). Binucleated cells were observed by imaging (indicated by white arrowheads) (Fig. 2F and fig. S13) (*43*). Flow cytometric analysis showed that ~9% of the total cell population was binucleated, with ~80% of these cells not in the active cell cycle (Fig. 2F, left, and figs. S13 and 14).

### Protein-rich biomaterials can be extracted from mammary tissue

**Fig. 3.**
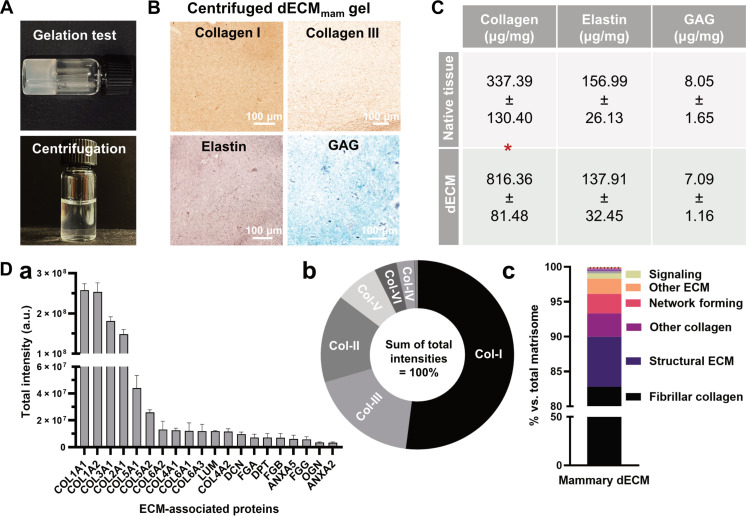
dECM from mammary tissue for light-based printing applications. (**A**) The final dECM_mam_ preserves self-gelling capacities (90° gelation test) postneutralization and after centrifugation (8000 rpm, 7 min) to obtain a transparent dECM_mam_ solution for light-based printing. (**B**) Histological sections of (centrifuged) dECM_mam_ gels stained for collagens I and III, elastin (Verhoeff’s stain), and GAGs (Safranin-O). Scale bars, 100 μm. (**C**) Quantification of ECM proteins: collagen, elastin, and GAG. Means ± SD (*n* = 4 batches). *P* < 0.05, with significant differences between native and decellularized tissue indicated by an asterisk (*) in the table. (**D**) Proteomic analysis of the ECM batches postdecellularization using intensity-based label-free quantification (*n* = 3 batches): (a) Top 20 matrisome proteins by total signal intensity. (b) Relative abundance of identified collagen subtypes displayed as a fraction of total collagen signal, with (c) classification according to the matrisome database (*46*). Fibrillar collagens were the most abundant in the dECM. a.u., arbitrary units.

To establish a bioactive base material for bioprinted mammary scaffolds, tissue was harvested from lactating bovine udders and decellularized (fig. S15A). Hughes’ work (*44*) and our histological staining demonstrate the resemblance of the bovine mammary gland to the human mammary gland, particularly with its collagen-rich stroma (fig. S15B). In addition, fresh bovine tissue was available in large quantities, which is crucial given the low yield of decellularized tissue dry mass of ~4% (fig. S15C). After decellularization, fat, nuclei, and double-stranded DNA were successfully removed (fig. S15, D to F). The mammary dECM’s (dECM_mam_) self-gelling properties were confirmed by a 90° thermal gelation test pre- and postcentrifugation, and gelation kinetics were assessed by thermal rheology (Fig. 3A and fig. S15F). All three independent dECM_mam_ batches formed comparable sigmoidal gelation curves (4° to 37°C) and reached a plateau in about 28 min, in line with previously reported studies (figs. S15F and S16) (*29*). Milk-derived MECs were encapsulated in thermally gelled dECM_mam_ and Matrigel, with matched rheological properties, and both materials successfully supported MEC survival (fig. S16).

For VP, the resin should be transparent to allow for light penetration and reduced scattering; hence, the dECM_mam_ was further processed by centrifugation (fig. S17, A and B) (*24*). This step removed undigested particles while preserving both high and low molecular weight proteins, as confirmed by SDS–polyacrylamide gel electrophoresis (fig. S17C). Our dECM_mam_ extraction protocol for light-based printing applications preserved key ECM components such as collagens, elastin, and glycosaminoglycans (GAGs), confirmed by the comparison of the native tissue to the decellularized tissue and ECM gels, following trends reported in the literature (Fig. 3, B and C, and fig. S18) (*29*).

For a better understanding of the native mammary ECM, the residual protein content of the dECM_mam_ was determined. Mass spectrometry (MS)–based proteomic analysis of the dECM_mam_ powders prepepsin digestion identified 1321 proteins, of which 131 were classified as ECM or ECM-associated (Fig. 3Da and fig. S19). Collagens are essential structural and functional ECM components found in the mammary tissue, and, in line with previous studies, collagen type 1 (Col-I; 52% of total collagen signal), Col-III (18%), Col-II (15%), Col V (7%), Col VI (4%), and Col-IV (3%) were the most abundantly identified collagens in the dECM_mam_ by total signal intensity (Fig. 3Db and fig. S20A) (*45*). Identified proteins were grouped according to functional classifications derived from the matrisome database (*46*). Fibrillar collagens (i.e., collagens I, II, and III) were the most predominant ECM components, crucial for maintaining 3D tissue structure (Fig. 3Dc and fig. S20, B and C). To cross-check the dECM_mam_ with other human tissues relevant for tissue engineering, a hierarchical clustering analysis of the 131 matrisome proteins was performed and compared to a draft map of the human proteome (*47*). The dECM_mam_ showed similarities especially with ectoderm-derived tissues (i.e., epithelial and nerve tissues) (fig. S21).

### VP dECM scaffolds mimic human breast architecture and biophysical properties

**Fig. 4.**
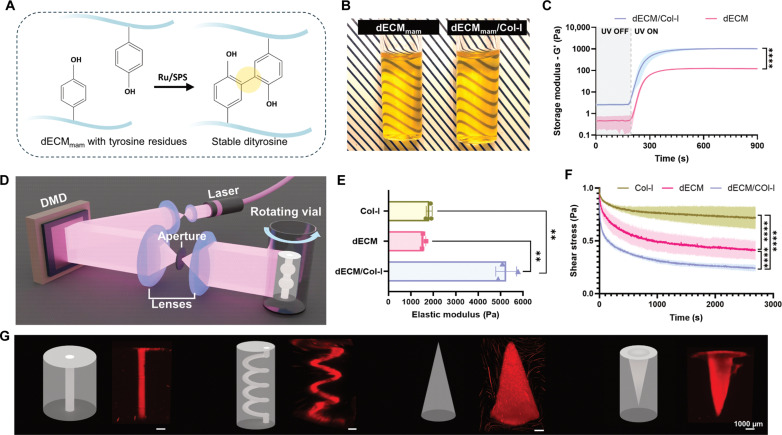
High-resolution VP of dECM_mam_ for breast tissue engineering applications. (**A**) The Ru/SPS photoinitiator system allows for the formation of dityrosine bonds upon exposure to 405-nm laser light. (**B**) Printing vial filled with cooled dECM_mam_ photoresin mixed with Col-I and Ru/SPS ready for light-based bioprinting. (**C**) Photorheology of the dECM_mam_ (40 mg/ml) and dECM_mam_/Col-I (40 + 4 mg/ml) resins demonstrating a rapid gelation response upon exposure to 405-nm light. *P* < 0.0001 for the plateau storage moduli. UV, ultraviolet. (**D**) Schematic of the VP set up comprised of a laser light source, a digital micromirror device (DMD), an aperture, lenses, as well as a printing vial mounted on a rotation stage, which allows for the creation of complex 3D shapes. (**E**) Elastic moduli of printed and fully postcured constructs with Col-I, dECM_mam_, and dECM_mam_/Col-I (means ± SD, *n* = 3 technical replicates, *P* < 0.01). (**F**) Printed dECM_mam_/Col-I constructs show slower stress relaxation responses, more closely reflecting the viscoelastic behavior of the native mammary tissue (*67*). *P* < 0.0001 indicated with asterisks for the measured equilibrium stress. (**G**) STL files depicted in gray and printed perfusable shapes [spiral and channel perfused with rhodamine acrylate (red)–labeled gelatin methacrylate] and resolution tests (cone and inverted cone) printed with rhodamine-stained dECM_mam_/Col-I resin. Scale bars, 1000 μm.

To transform the dECM_mam_ into a light printable material, the ruthenium/sodium persulfate (Ru/SPS) photoinitiator was added. Ru/SPS allowed the cross-linking of tyrosine residue–carrying proteins found in dECM_mam_ by stable dityrosine cross-link formation upon light exposure, without additional chemical modification (Fig. 4A).

Previous studies suggested that printing fidelity is strongly dependent on material and Ru/SPS concentration (*26*, *48*). To investigate the photoinitiator’s effects on the material, Ru/SPS concentrations and ratios from 1:1 to 1:20 were iteratively tested by photorheology using a 405-nm light source (fig. S22). Increased photoinitiator concentrations did not correlate with faster cross-linking speeds (i.e., faster gel formation) or a higher storage modulus. This may be attributed to a limited availability of reactive tyrosine groups, whereby increased photoinitiator concentrations would not enhance cross-linking. Furthermore, higher photoinitiator levels could increase photoabsorption, which hinders light penetration and cross-linking efficiency. The gelation was further tunable by the ratio of the two photoinitiator components (Ru and SPS). A 1:10 ratio of 0.5 mM Ru to 5 mM SPS demonstrated the optimal balance of stiffness, cross-linking speed, and minimal photoinitiator concentrations to avoid cytotoxicity on cells (fig. S22A).

To evaluate the effects of material concentrations, the Ru/SPS ratio was fixed at 0.5/5 mM, and dECM_mam_ varied from 20 to 60 mg/ml. Beyond 40 mg/ml, an additional material did not enhance the storage modulus (maximum, ~120 Pa) but slowed down the cross-linking response (fig. S22B). This could be explained by particle precipitation. For mechanical property tuning of the dECM_mam_ at 40 mg/ml, Col-I was added at concentrations between 1 and 5 mg/ml. The addition of Col-I (4 mg/ml) produced a plateau/maximum storage modulus of ~1000 Pa, but further increases to 5 mg/ml reduced the modulus back to 450 Pa (fig. S22, C and D). The final photoresin material optimized for mammary scaffold bioprinting consisted of dECM_mam_ (40 mg/ml) and Col-I (4 mg/ml) combined with 0.5 mM Ru and 5 mM SPS, with a refractive index of 1.345 (Fig. 4B and fig. S23, A to F). This formulation allowed to increase viscoelasticity and stiffnesses (Fig. 4, E and F, and fig. S23B).

After resin optimization, dECM_mam_/Col-I spiral, cone, and inverted cone models were VP using a light dose of 1250 to 1350 mW/cm^2^ for 120 s, achieving a positive resolution of 300 μm (Fig. 4G and figs. S24 and S25). Unlike constructs made from Col-I or dECM_mam_ alone, which lost their integrity within a month (fig. S23, B to F), the dECM_mam_/Col-I constructs maintained their shape and mechanical properties for over 1 month. These stable and high-fidelity structures offer a solid foundation for further exploration in mammary tissue modeling.

### Volumetrically printed (VP) dECM_mam_/Col-I alveoli support milk protein production in vitro

To test biocompatibility and cell adhesion, we printed cylinders on which our milk MECs were seeded. The biocompatibility of the constructs was confirmed by high cell viability (>90%) and the ability of the MECs to completely cover the printed surface within 7 days (fig. S26, A and B). Lactate dehydrogenase assays over a 72-hour period confirmed that the scaffolds were noncytotoxic (fig. S26C). The stiffness of seeded cylinders was constant over 1 week, demonstrating the stability and mechanical integrity of printed constructs in the presence of cells (fig. S26D). Five-day-old milk MEC layers maintained their barrier function on dECM/Col-I hydrogels and effectively blocked rhodamine diffusion into the gel, unlike the cell-free control (fig. S27).

**Fig. 5.**
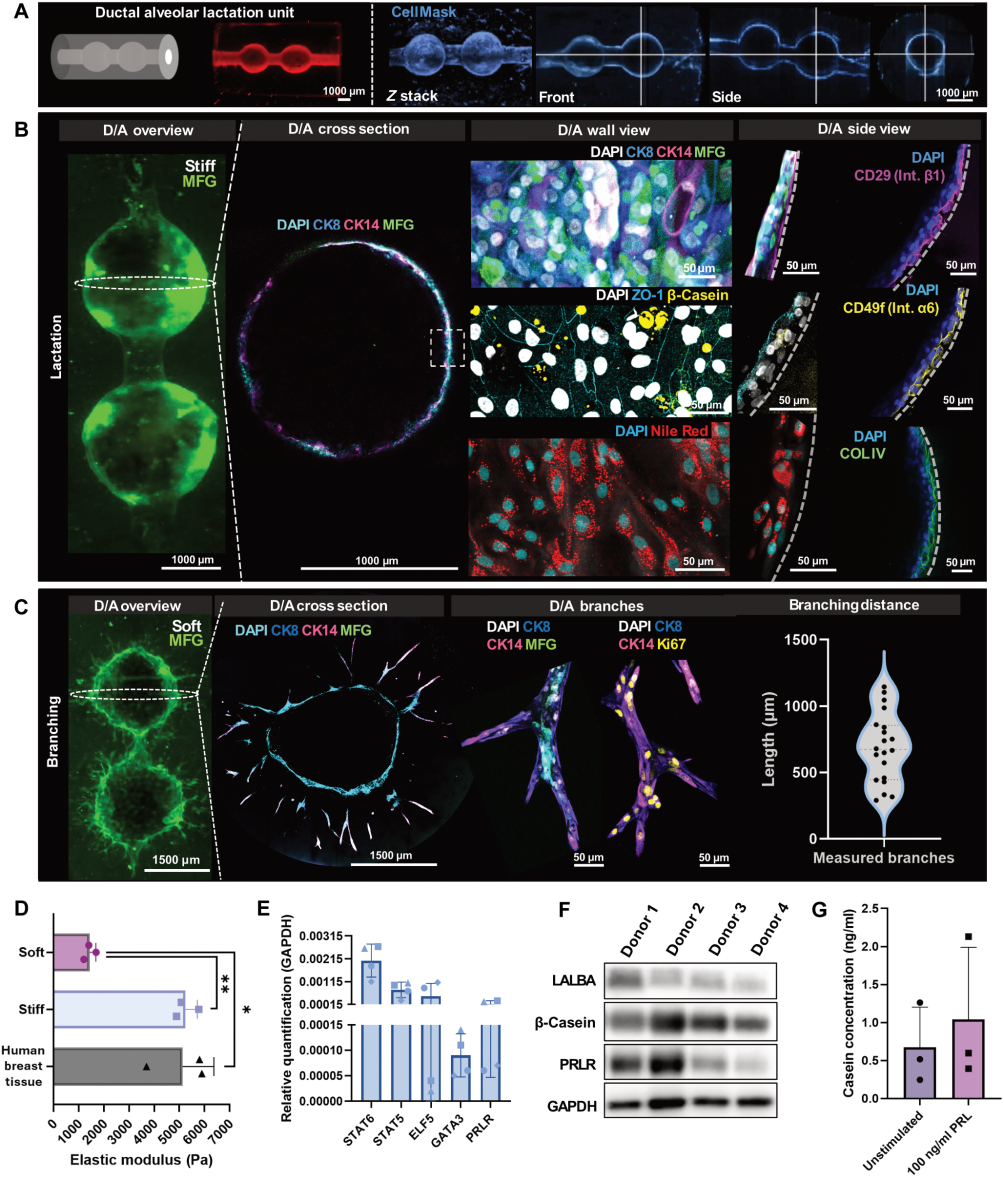
VP dECM_mam_/Col-I alveoli support milk MEC function in vitro. (**A**) Perfusable ducal alveolar units were generated using computer-aided design (gray; left). D/A units were perfused with rhodamine-labeled Gel-MA and CellMask-labeled cells (at day 7) to show homogenous distribution of injected material and even spread of cells in the printed constructs. The white cross indicates the sections where the displayed image slices were taken from the 3D stack. (**B**) Immunofluorescent staining of “stiff” VP alveoli with milk MECs at day 7 after seeding (left, lightsheet imaging; right, confocal imaging). Confocal images of the D/A units stained with CK8, CK14, MFG, β-casein, Nile Red (lipids), and ZO-1 to demonstrate the secretory capacities. CD29 and CD49f were expressed basally and ZO-1 apically as seen in the side views. (**C**) Cells on the softer construct were stained with CK8, CK14, MFG, and Ki67 to show branch elongation and proliferation. (**D**) Elastic modulus of the softer (VP only) and stiffer (VP and postcuring) dECM_mam_/Col-I gel matched with the measurements done for healthy human breast tissue (means ± SD, *n* = 3 technical replicates for printed scaffolds, *n* = 3 biological replicates for human tissue). (**E**) RT-qPCR of selected lactation relevant genes expressed by milk MECs on 3D-printed constructs (means ± SD, *n* = 4 biological replicates). (**F**) Western blot of milk-associated proteins (β-casein and LALBA), PRLR, and housekeeping gene (GAPDH) expression after prolactin stimulation of milk MECs (four donors) seeded in the printed D/A scaffolds. (**G**) Enzyme-linked immunosorbent assay showing β-casein secretion into the media with and without the addition of prolactin and compared to human milk (means ± SD, *n* = 3 biological replicates, donors). *P < 0.05, **P < 0.01.

For lactation experiments, perfusable duct/alveoli (D/A) units were designed with a channel diameter of ~800 μm and an alveoli diameter of ~1500 μm. To test perfusion, the D/A units were injected with rhodamine-labeled gelatine methacrylate (Fig. 5A, left). Next, milk MECs were postseeded by injection into the hollow D/A unit tube. The cells formed a continuous monolayer on the printed surface and expressed CK8, CK14, and tight junction maker ZO-1 on the apical side of the epithelial layer (Fig. 5, A and B). Positive milk fat globule (MFG) and β-casein stainings showed droplet formation and milk protein production on our scaffolds (Fig. 5, B and C). Nile red staining reconfirmed the presence of lipid droplets within the epithelial lining of the D/A unit (Fig. 5B and fig. S28A). Furthermore, the cells expressed CD29 and CD49f toward the basal side and deposited Col-IV on the printed D/A units (Fig. 5B, right, and fig. S28B). The broader applicability of our D/A units in mammary tissue engineering was assessed by seeding MCF10-A cells, bovine tissue–derived MECs, and bovine milk-derived MECs. Cell adhesion, epithelial layer formation, CD49f expression, Col-IV deposition, and the presence of lipids (Nile Red staining) were observed (fig. S29).

To understand the effect of material stiffness on cell behavior in our printed constructs, stiff (5000 Pa) and soft (1300 Pa) hydrogels were printed by modulating the light exposure (Fig. 5, B and C). Cells on the softer (on the scale of Matrigel) scaffolds developed branching structures that penetrated the hydrogel with CK8- and CK14-positive MECs and MFG expression. Over a 7-day culture period, these branches grew to ~750 μm in length and continued to display proliferative potential (Ki67^+^ cells) (Fig. 5C). In the stiff gels [fabricated to match measured human breast tissue samples (3 to 5 kPa), also shown in Fig. 4E], the cells lined the printed hollow D/A units but were unable to escape the predefined shape. These results showcased our platform’s tunability and potential not only for lactation but also for possible other applications such as in mammary tissue dynamics studies.

For a better characterization of our lactation model in the context of signaling pathways, real-time quantitative polymerase chain reaction (RT-qPCR) was used to assess the expression of pregnancy- and lactation-associated genes, including STAT5, STAT6, E74-like transcription factor 5 (ELF5), GATA binding protein 3 (GATA3), and PRLR (Fig. 5D). STAT5 and STAT6 transcription factors mediate prolactin and cytokines during MEC differentiation and lactation (*49*). ELF5 and GATA3 are involved in alveolar development during lactogenesis, while PRLR facilitates hormonal signaling (*50*, *51*). Milk MECs seeded onto mammary D/A units expressed studied lactation-associated genes, and follow-up Western blot analysis confirmed the presence of activated phosphorylated STAT5 (fig. S30). PRLR expression assessed by Western blot was in line with the RT-qPCR results in showing biological differences in the expression of this receptor (Fig. 5F). β-Casein and lactalbumin (LALBA) bands reconfirmed the presence of milk proteins (Fig. 5E and fig. S31). No correlation between PRLR expression levels and the expression of studied milk proteins was observed in these experiments. To assess the secretion of milk components, we performed an enzyme-linked immunosorbent assay on the culture medium (Fig. 5F). Here, casein was detected with and without prolactin stimulation.

## DISCUSSION

While mammary gland research was previously heavily focused on in vivo studies and organoid work, recent advances in light-based 3D printing offer exciting possibilities for in vitro breast tissue models. In this context, we developed a light-responsive biomimetic material based on decellularized mammary tissue and introduced VP to create lactating tissue scaffolds. Our printed D/A lactation units promoted adhesion, survival, proliferation, and the function of MECs. Nontraditional MEC sources such as human milk offer significant opportunities for lactation studies, and isolated milk MECs were able to synthesize milk proteins such as β-casein, LALBA, and MFGs in the printed lactation units.

MECs were isolated from human breast milk, and our flow cytometric analysis showed that most cells shed into the milk were nonepithelial, with MECs compromising only ~11% of the total cell population. Among these shed MECs, only luminal MECs were detected (~8% mature luminal cells and 3% luminal progenitors of total cells, with no detectable basal-like cells), which was consistent with previously published single-cell RNA-seq studies (*30*, *32*). In contrast, during cell expansion, we observed a shift in cell markers, where cells were predominantly epithelial and expressed both luminal (progenitor CD49f^+^/EPCAM^+^ and mature CD49f^−^/EPCAM^+^) and basal-like (CD49f^+^/EPCAM^−^) MEC characteristics. The emergence of basal-like (CD49f^+^/EPCAM^−^) cells in culture may result from mechanisms such as trans-differentiation caused by culture-induced plasticity, differentiation of progenitor cells, or selective advantages of 2D culture conditions that may promote the expansion of this otherwise rare cell population (*33*, *40*). Further in vitro characterization of milk MECs is required to fully clarify the basal phenotype of CD49f^+^/EPCAM^−^ cells, which has not yet been definitively established. Milk MECs were expandable while preserving their epithelial phenotype and PRLR expression. Further investigations could explore whether this receptor is expressed by milk MECs in short, intermediate, or long isoform, as this could provide valuable insights into its functional role in milk production and regulation (*52*). Under the protocol used in this study, milk MEC expansion was limited to three passages. This restricted higher experimental throughput and larger milk production. Here, future efforts in milk MEC culture should consider 3D expansion techniques. Organoid research suggests that this approach enhances cell plasticity and adaptability, which can lead to more robust cell growth (*53*). Furthermore, the expansion of cells in their natural organization, with apical and basolateral domains, holds promise for effective restructuring into two distinct layers (luminal on top of basal MECs) after seeding in 3D-printed scaffolds (*54*, *55*). The in vivo–like organization of the epithelium is crucial for the directional transport of milk components and the response to lactation hormones (prolactin and oxytocin). Here, the effects of different media components on (milk) MEC populations and lactation potential should be thoroughly evaluated, in line with existing literature, to improve the lactation potential and hormone response of milk MECs (table S2) (*35*, *56*, *57*). In addition, Wnt signaling and ErbB receptor pathway activation should be targeted in the future by the addition of R-spondin and neuregulin-1 in the medium to enhance MECs’ self-renewal and further promote bilayer organization, respectively (*58*).

We established dECM_mam_ gels as a bioactive base material for mammary tissue engineering applications and successfully printed biomimetic mammary tissue scaffolds. To provide optimal support to the cells from the moment they contact the scaffold, we could manually introduce BM proteins rather than relying on the cells’ own secretion. Laminin and Col-IV are essential components of the BM, and laminin produced by basal MECs is key for progenitor self-renewal and basal-apical polarity (*59*, *60*). Thus, the alveolar channel could be coated with a thin layer of Col-IV and laminin 111 and cross-linked with Ru/SPS upon light exposure.

On D/A units, all donor cells maintained their PRLRs that made them suitable for milk secretion in vitro. Building on this proof of concept, hormone concentrations can be further optimized, and oxytocin can be added to test for basal cell contraction upon polarization, to reach efficient lactation of milk MECs in engineered tissue models (*57*, *61*)*.* To move toward a streamlined and dynamic system where hormones and nutrients can be readily modulated and milk can be continuously collected, the VP D/A units can be integrated with an automatic gel flipper for cell seeding and peristaltic pump for perfusion (*62*). Perfusion will not only enhance automation and standardization, but the use of multiple D/A units in parallel would increase throughput when testing MECs’ behavior from several donors under different hormonal conditions. Furthermore, the pumping system would allow for not only continuous but also pulsatile flow patterns to recapitulate the fluctuating hormonal levels found in vivo and breastfeeding intervals.

Lactation potential among donor-derived cells varied in our study, where certain samples exhibited higher secretory capacity than others. Here, the sample collection process should be reconsidered. Observed differences may have arisen not only from biological variability but also from sample collection timing, as milk composition fluctuates both throughout the lactation period and at different times of day (*54*). An increased sample size with set lactation time points or longitudinal studies across multiple pumping time points could offer more comprehensive and statistically relevant insights into the characteristics of MECs throughout breastfeeding. The identification of “high potential” donor cells during identified optimal time frames would present a viable approach to maximize the in vitro–produced milk yield for further quantitative and qualitative milk analysis.

The materials and cells presented in this study could be adapted for multiple research questions and in vitro models. In future studies, a combination of natural materials such as dECM or Col-I with synthetic materials (e.g., polyacrylamide or polyethylene glycol) could further reduce (mechanical) variations of the hydrogels and offer more flexibility when tuning stiffness or viscoelasticity. This could allow us to model different states of breast development and the study of MECs-ECM interactions. In addition, the printing resolution can be improved by the incorporation of a free radical inhibitor, such as 2,2,6,6-tetramethyl-1-piperidinyloxy, to enhance control over the polymerization process (*63*). This would allow for studies on the effects of smaller feature sizes on milk MECs’ function and the optimization of surface topography for increased milk production. Furthermore, our materials have both photocrosslinkable and thermal gelation properties. This dual capacity could allow the use of dECM_mam_/Col-I as a thermally gelled coating in hollow fiber bioreactors, to achieve the upscaling of in vitro milk component production for infant nutrition down the line.

Our VP mammary model, along with its individual components such as milk MEC and dECM, provides a valuable addition to existing animal and organoid models for lactation research. Notably, the acellular nature of VP scaffolds also facilitates their broader dissemination within the field, where interested researchers could seed various cells, including MECs from healthy or diseased tissue. This flexibility holds great promise for advancing both basic and translational research in mammary gland biology and lactation.

## MATERIALS AND METHODS

### Bovine tissue collection, decellularization, and ECM extraction

Fresh bovine lactating udders were procured from the local slaughterhouse and transported to the lab immediately. The alveolar tissue was cut into ~300-g pieces washed in a 5% penicillin/streptomycin (Pen/Strep; Gibco, 15140122) deionized water solution and then frozen at −20°C for short-term storage and at −80°C for long-term storage. For decellularization, previously published protocols for decellularization of other tissues were adapted (*64*). Briefly, the semifrozen tissue was cut by hand into cubes of ~1.5 mm^3^ and washed in Milli-Q water for 2 hours. The tissue pieces were washed with a 1% SDS (Sigma-Aldrich, 75746) for 2 hours and then decellularized in fresh 1% SDS (Sigma-Aldrich, 75746) for 16 hours (overnight) at room temperature (RT), adding 2% Pen/Strep. The pieces were then transferred to isopropanol for 5 hours, with frequent changes, before thoroughly washing with several Milli-Q water changes. All steps were performed on a magnetic stir plate at 250 rpm. The decellularized tissue was then freeze dried (72 hours) and cryomilled (Retsch) into a fine powder. The powder was enzymatically digested with pepsin (1 mg/ml; Sigma-Aldrich, P7012-250MG) in 0.1 M HCl for 48 hours on a magnetic stir plate (150 rpm) and then brought to neutral pH on ice (300 rpm). The neutralized dECM_mam_ was centrifuged at 8000 rpm for 7 min, freeze dried, and stored at −20°C.

### Human tissue collection and preparation for mechanical measurements

Ethics approval for the study was obtained (Kantonale Ethikkomission, 2022-01844), and tissue samples were collected after written informed consent. The samples were transported to the lab on ice, where adipose tissue was dissected from the epithelial tissue. Mechanical measurements were performed exclusively on the epithelial tissue, with round sections being extracted using a biopsy punch.

### DNA, collagen, elastin, and glycaminoglycan quantification

Decellularized tissue pieces were randomly chosen, and DNA was quantified using the PureLink Genomic DNA Mini Kit (Invitrogen, K182001). The final concentration of DNA in the samples was measured using a NanoDrop. Collagen content was measured using the QuickZyme Collagen assay kit (QuickZyme Biosciences), a Blyscan Sulfated GAG Assay kit (Biocolor, B3000) was used for the GAGs, and an Elastin Assay kit (Biocolor, F4000) was used for elastin. The providers’ protocols were followed for these experiments.

### Sample preparation for proteomic analysis

Samples were taken from *n* = 3 bovine dECM batches (four bovine lactating udders pooled into one batch). Two milligrams of lyophilized material from each batch was individually combined with 100 mg of 3-mm glass beads in 1.5-ml Safe-Lock tubes (Eppendorf) and homogenized in 6 M guanidine hydrochloride (200 μl/mg) and 100 mM ammonium bicarbonate at power 8 for 1 min (Bullet Blender, Model BBX24, Next Advance Inc.). Each sample was vortexed (power 5) at RT (25°C) overnight. Homogenate was spun at 18,000*g* (4°C) for 15 min, and the supernatant was collected as the soluble ECM fraction. Pellets were then treated with freshly prepared hydroxylamine buffer (1 M NH_2_OH-HCl, 4.5 M Gnd-HCl, 0.2 M K_2_CO_3_, and pH adjusted to 9.0 with NaOH) at 200 μl/mg of the starting tissue dry weight. Samples were homogenized at power 8 for 1 min and incubated at 45°C with shaking (1000 rpm) for 4 hours. Following incubation, the samples were spun for 15 min at 18,000*g*, and the supernatant was removed and stored as the insoluble ECM fraction at −80°C until further proteolytic digestion with trypsin. All fractions were subsequently subjected to overnight enzymatic digestion with trypsin (1:100, enzyme:protein ratio) using a filter-aided sample preparation approach and desalted during Evotip loading (described below) (*65*).

### Liquid chromatography–tandem MS analysis

Digested peptides were loaded onto individual Evotips following the manufacturer’s protocol and separated on an Evosep One chromatography system (Evosep, Odense, Denmark) using a Pepsep column (150 μm in inner diameter, 15 cm) packed with ReproSil C18 1.9 μm, 120-Å resin. Samples were analyzed using the instrument’s default “30 samples/day” liquid chromatography gradient. The system was coupled to the timsTOF Pro mass spectrometer (Bruker Daltonics, Bremen, Germany) via the nanoelectrospray ion source (Captive Spray, Bruker Daltonics). The mass spectrometer was operated in Parallel Accumulation Serial Fragmentation mode. The ramp time was set to 100 ms, and 10 PASEF tandem MS (MS/MS) scans per topN acquisition cycle were acquired. MS and MS/MS spectra were recorded from mass/charge ratio 100 to 1700. The ion mobility was scanned from 0.7 to 1.50 Vs/cm^2^. Precursors for data-dependent acquisition were isolated within ±1 Thomson and fragmented with an ion mobility–dependent collision energy, which was linearly increased from 20 to 59 eV in the positive mode. Low-abundance precursor ions with an intensity above a threshold of 500 counts but below a target value of 20,000 counts were repeatedly scheduled and otherwise dynamically excluded for 0.4 min.

### Global proteomic data analysis

Data were searched using MSFragger via FragPipe v21.1. Precursor tolerance was set to ±15 parts per million, and fragment tolerance was set to ±0.08 Da. Data were searched against UniProt restricted to *Bos taurus*, with added common contaminant sequences (37,606 total sequences). Enzyme cleavage was set to semispecific trypsin for all samples. Fixed modifications were set as carbamidomethyl (C). Variable modifications were set as oxidation (M), oxidation (P) (hydroxyproline), dioxidation (P), deamidation (NQ), Gln → pyroGlu (N-term Q), and acetyl (peptide N-term). Label-free quantification was performed using IonQuant v1.10.12 with match-between-runs enabled and default parameters. Soluble and insoluble ECM fractions were searched separately and merged after database searching. Results were filtered to 1% false discovery rate at the peptide and protein levels.

### Resin preparation

Col-I (Advanced Biomatrix, 5005) was resuspended at 10 mg/ml in phosphate- and glucose-rich buffer and left to dissolve overnight in the fridge. The lyophilized dECM_mam_ (80mg/ml) was resuspended in 1× phosphate-buffered saline (PBS) on ice as stock solutions for resin preparation. A total of 0.5 mM RU and 5 mM SPS were added to the freshly prepared resin [Col-I (4 mg/ml) and dECM_mam_ (40 mg/ml)] in the dark on ice by thoroughly pipetting up and down using a viscous pipet.

### (Photo)rheology

All rheological experiments were performed using an Anton Paar MCR 302e rheometer with a 20-mm parallel plate geometry. Shear measurements were performed at a shear rate of 2% and a frequency of 1 Hz, with acquisition intervals of 10 s. To maintain sample integrity and prevent drying during testing, a wet tissue paper was added in the chamber. Seventy-six microliters of the sample was loaded onto the rheometer, with a gap distance of 0.2 mm.

For evaluation of thermal gelation and temperature-dependent rheological properties of the pure dECM, the lyophilized dECM powder was dissolved on ice on the same day in cooled 1× PBS and loaded onto the 4°C cooled stainless steel floor of the rheometer. During measurement, the temperature increased to 37°C after 10 min.

For photorheology of the ECM-based resins, a glass floor was used. The rheometer was combined with the Omnicure Series1000 lamp (Lumen Dynamics) with sequential 400 to 500 nm and narrow 405-nm band-pass filters (Thorlabs). dECM_mam_-based photoresins were prepared in the dark on ice, as previously described. Measurements were left to proceed in the dark for 3 min before irradiating the sample with 405-nm light at (60%) 10 mW/cm^2^ intensity.

### Mechanical testing

Compression tests were performed on a TA.XT Texture Analyzer (Stable Micro System) with a 500 g of load cell. Samples were placed between the compression plates, and a preload of 0.2 g was applied to ensure full contact of the samples with the plates. Samples were then compressed to 15% strain at 0.01 mm/s. Loading and unloading curves were recorded, and the compressive modulus was calculated by linear fitting the first 3% of the stress-strain curve.

### Volumetric printing

3D printing was carried out using the open format volumetric printer (Readily3D SA, CH). The performance of the mammary photoresin was assessed with the built-in software feature for light dose tests to study the resin’s behavior in the printer’s settings, light source, and path. Before printing, dose tests were performed by projecting circles (ɸ = 1 mm), with increasing light intensity (784 to 2304 mJ/cm^2^), onto a 1-mm path length cuvette filled with the cooled dECM_mam_-based photoresin (fig. S16). For printing, 1 ml of resin was filled in the dark on ice in 10-mm Pyrex glass printing vials. To remove the noncross-linked resin postprinting, the constructs were washed in cold 1× PBS by gently pipetting up and down. Stereolithography (STL) files for printing were created using Fusion 360 (Autodesk, Fusion 2.0.20494 x86_64). Printed constructs were postcured in an ultraviolet box to increase the stiffness of the constructs and match the mechanical properties to the one measured human breast tissue.

### Milk collection, mammary epithelial isolation, and culture

Human donors were recruited in line with Swiss Ethics guidelines, and written informed consent was obtained from all participants by trained personnel (Kantonale Ethikkomission, 2022-02012). Human milk collected by a breast pump was immediately transported to the lab on ice. Cells were isolated by centrifugation using a previously published protocol by Twigger *et al.* (*32*). Briefly, cooled 1× PBS was added to the freshly collected milk (1:1 ratio) and centrifuged for 20 min, 780*g* at RT. The pellet was transferred to a fresh falcon (15 ml) and washed three times in 1× PBS with 1% antianti (Gibco, 15249-062) by centrifuging at 480*g*, 5 min at 4°C. The pellet was then resuspended in Mammary Epithelial Cell Growth Medium (MECGM) (PromoCell, C-21010) with 10 μM forskolin (Sigma-Aldrich, F6886), 3 μM Rock-I (Y-27632) (Sigma-Aldrich, Y0503), and 1% Pen/Strep (Gibco, 15140122). Cells were plated on with Matrigel (Corning, 11573620)–coated T25 polystyrene cell culture flasks. The milk MECs were left to attach for 5 days before changing the medium to MECGM with 10 μM forskolin, 0.5% human serum (Sigma-Aldrich, H4522), and 1% Pen/Strep (composition adapted from previously established protocols). For further cell passaging (P1 to P3), cells were cultured in MECGM with 10 μM forskolin, 5% human serum, and 1% Pen/Strep. Cells were split at 80 to 90% confluency using TryplE (Gibco, 12604021) at 37°C and at 5% O_2_ and reseeded at a density high density of 6000 cells/cm^2^ for expansion.

### Construct seeding and culture

Two to 4 million cells/ml were perfused into the printed alveoli. The construct was placed in an empty 24-well plate and gently rotated by hand in an incubator for 50 min. During the first 20 min, the plate was rotated 90° every 5 min. Next, the plate underwent two 180° rotations, followed by a 90° rotation and an additional 180° rotation. After this initial cell adhesion phase, the well was gently filled with culture medium and placed back in the incubator.

### Flow cytometry staining and analysis

Single-cell suspension was first incubated for 10 min at RT with BD Pharmingen Human BD Fc Block (1:50; BD, 564219) and then stained with antibody mix (table S2) in fluorescence-activated cell sorting (FACS) buffer (PBS with 2% fetal bovine serum and 0.5 mM EDTA) for 30 min at 4°C. The Zombie NIR Fixable Viability Kit (1:1000; BioLegend, 423105) in PBS was used to discern live from dead cells. Cells were fixed in 4% paraformaldehyde (PFA) for 10 min, then washed, and resuspended in FACS buffer before running the cells on a BD LSR Fortessa machine (BD FACSDiva8.0.1 software) and FlowJo v10 software. Gating strategies are detailed in the Supplementary Materials. The antibody list can be found in table S3.

### Immunohistochemistry and confocal imaging

Constructs and cells were fixed in 4% PFA for 8 hours or 20 min, respectively, before immunofluorescent staining. The constructs/cells were washed three times in PBS and blocked with 5% bovine serum albumin (BSA; MilliporeSigma) with 0.2% Triton X-100 (100 ml; Sigma-Aldrich, T8787) in PBS for 35 hours at RT. The constructs were then incubated with primary antibodies (table S3) in BSA-PBS overnight at 4°. On the next day, the samples were washed three times in PBS and incubated with the secondary antibody solution (table S4) and 4′,6-diamidino-2-phenylindole diluted in BSA-PBS for 2 hours at RT. Before imaging, samples were washed three times in PBS and imaged using an FVOlympus 3000 confocal microscope. Used antibodies can be found in tables S4 and S5.

### Western blotting

Milk MECs were grown on 3D-printed constructs for 2 days and were stimulated with prolactin for another 5 days before protein isolation. Cells were lysed in radioimmunoprecipitation assay buffer supplemented with protease inhibitors (Sigma-Aldrich, P1860-1ML, Burlington, Massachusetts, USA) and then centrifuged for 10 min at 12,000*g*. Total protein contents were calculated using Pierce 660 nm Protein Assay (Thermo Fisher Scientific, 22660, Waltham, Massachusetts, USA). Ten micrograms of proteins was mixed with NuPAGE Sample Reducing Agent (Invitrogen, NP0004, Waltham, Massachusetts, USA) and NuPAGE LDS Sample Buffer (Invitrogen, NP0007, Waltham, Massachusetts, USA) and denatured for 10 min at 80°C using a thermocycler. Samples were run on a NuPAGE 4 to 12%, bis-tris, 1.0 to 1.5 mm, Mini Protein Gel (Thermo Fisher Scientific, NP0321BOX, Waltham, Massachusetts, USA) and transferred onto a nitrocellulose membrane. The membrane was incubated overnight with primary antibodies (listed in table S6) at 4°C. Membranes were washed with PBS supplemented with 0.1% Tween 20 and incubated with a secondary horseradish peroxidase (HRP)–conjugated goat antirabbit or antimouse immunoglobulin G antibodies. The HRP signal was detected with WesternBright ECL HRP substrate (Advansta K-12045-D20, San Jose, California, USA) and imaged with a FUSION FX6 EDGE Imaging System (Witec, Sursee, Switzerland).

### RNA extraction and RT-qPCR

Milk MECs were cultured in two technical duplicates for each biological replicate on 3D-printed constructs for 5 days. Cells were incubated for 10 min in Buffer RLT - Lysis Buffer and then mixed with 70% ethanol before transfer to the RNeasy mini kit (QIAGEN, 74104) column to extract the total RNA. An A260/280 ratio between 1.8 and 2.1 was accepted as adequate quality for the RNA samples. One hundred nanograms of RNA was retrotranscribed with a GoScript Reverse Transcriptase kit (Promega, A5003, Madison, Wisconsin, USA). cDNA was diluted 1:5 with ribonuclease-free water. RT-qPCR was performed with GoTaq qPCR Master Mix (Promega, A6002, Madison, Wisconsin, USA). Reactions were run on a QuantStudio 3 96-well 0.1 ml Block Real-Time PCR System (Applied Biosystems, Waltham, Massachusetts, USA). Samples were analyzed using amplification and melting curves. Ct values were normalized to glyceraldehyde-3-phosphate dehydrogenase. All RT-qPCR primer pairs designed for this study were bought through Microsynth AG (Balgach, Switzerland) and can be found in table S7.

### Lightsheet microscopy

An axially scanned lightsheet microscope (MesoSPIM, V4) was used to image the VP constructs (*66*). The constructs were transferred to a 4-mm glass cuvette with Milli-Q water, mounted onto a custom 3D-printed sample holder, and submerged in a quartz chamber filled with Milli-Q water, mounted onto the MesoSPIM microscope stand. For imaging, a macro-zoom system (Olympus, MVX-10) and 2× air objective (Olympus, MVPLAPO1x) with adjustable zoom were used. Voltage adjustments using the electrically tunable lens were performed for each run. Step size was chosen from 5 to 50 μm.

### Quantification and statistical analysis

Experiments were conducted with three or more replicates, as specified. The sample size (*n*) for each experiment is provided in the corresponding figure legends. Data analysis was performed using Excel (Microsoft 365 MSO Version 2412 Build 16.0.18324.20092 64-bit), MATLAB R2018a (9.4.0.813654), GraphPad Prism (version 10.4.0 for Windows), and Fiji/ImageJ 1.54f (Java 1.8.0_332). Unless otherwise stated, data are presented as means ± SD, and statistical significance was assessed using a paired *t* test. *P* < 0.05 was considered statistically significant. Only significant results are shown in the figure panels except if otherwise specified in the figure legends.

## Supplementary Materials

### This PDF file includes:

Figs. S1 to S31 Tables S1 to S7 Supplementary Methods References
