# Supplementary material for: Volumetric printed biomimetic scaffolds support in vitro lactation of human milk-derived mammary epithelial cells
Source: Sci Adv. 2025 Jun 4;11(23):eadu5793. doi: 10.1126/sciadv.adu5793 (PMC12136022; doi:10.1126/sciadv.adu5793)
Supplement: Supplementary file 1 — Figs. S1 to S31 Tables S1 to S7 Supplementary Methods References [file sciadv.adu5793_sm.pdf]

Science Advances
AAAS

Supplementary Materials for

Volumetric printed biomimetic scaffolds support in vitro lactation of human milk-derived mammary epithelial cells

Amelia Hasenauer *et al.*

Corresponding author: Marcy Zenobi-Wong, marcy.zenobi@hest.ethz.ch

Sci. Adv. **11**, eadu5793 (2025)
DOI: 10.1126/sciadv.adu5793

This PDF file includes:

Figs. S1 to S31
Tables S1 to S7
Supplementary Methods
References

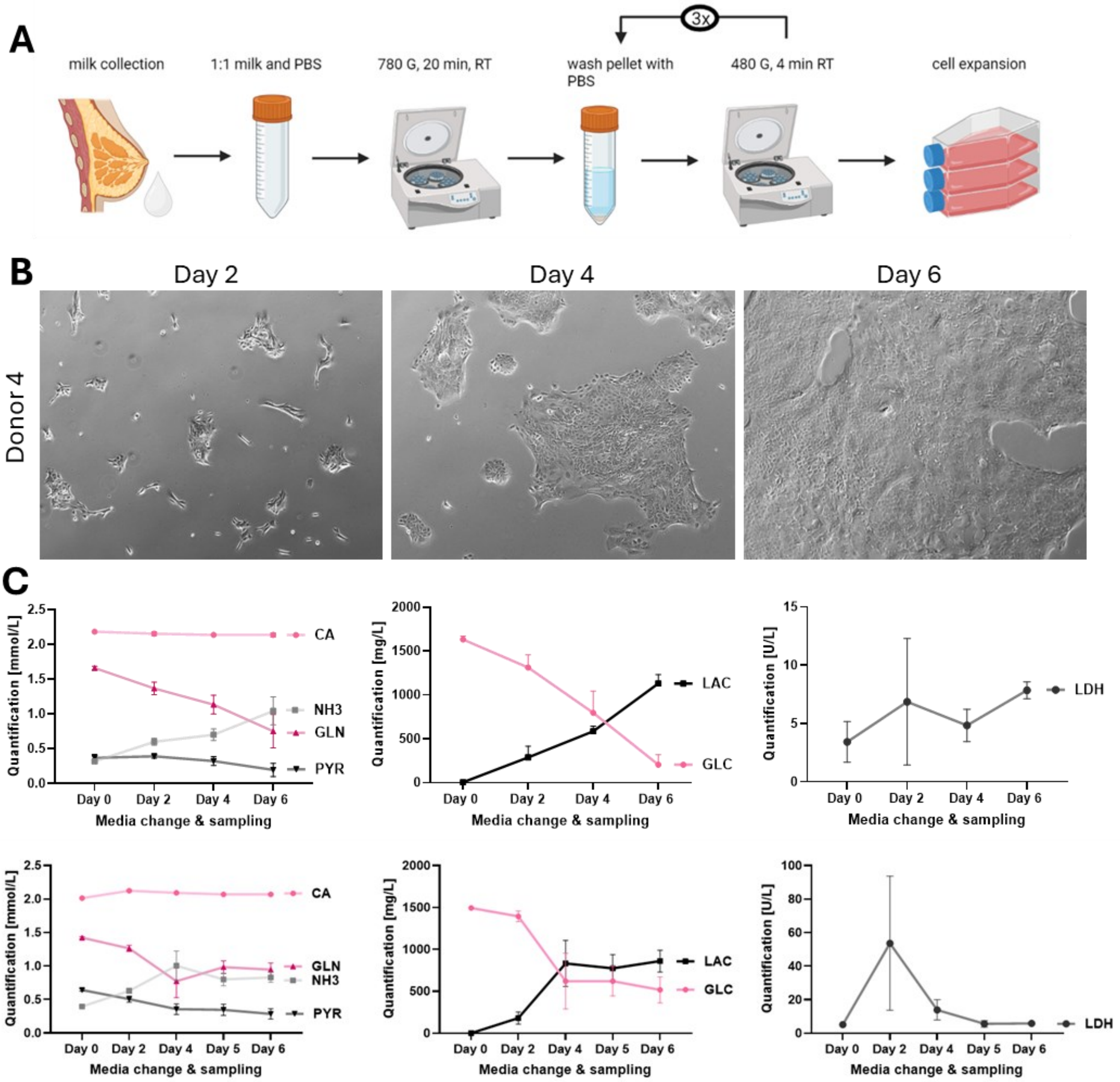

Figure S1: Milk cell isolation and culture

A) Human milk cells were isolated based on centrifugation. B) Brightfield images of isolated milk MEC expansion over the time span of one week and corresponding C) Analysis of the media throughout the culture period. Levels of calcium, ammonia, glutamine, pyruvate, lactate, glucose and lactate dehydrogenase were measured using the Cedex media analyzer. As cell numbers increased, media changes every two days (top) were adjusted to daily media changes from day 4 onward to compensate for glucose consumption and lactate secretion (bottom) (mean ± SD, n = 4 independent cell cultures).

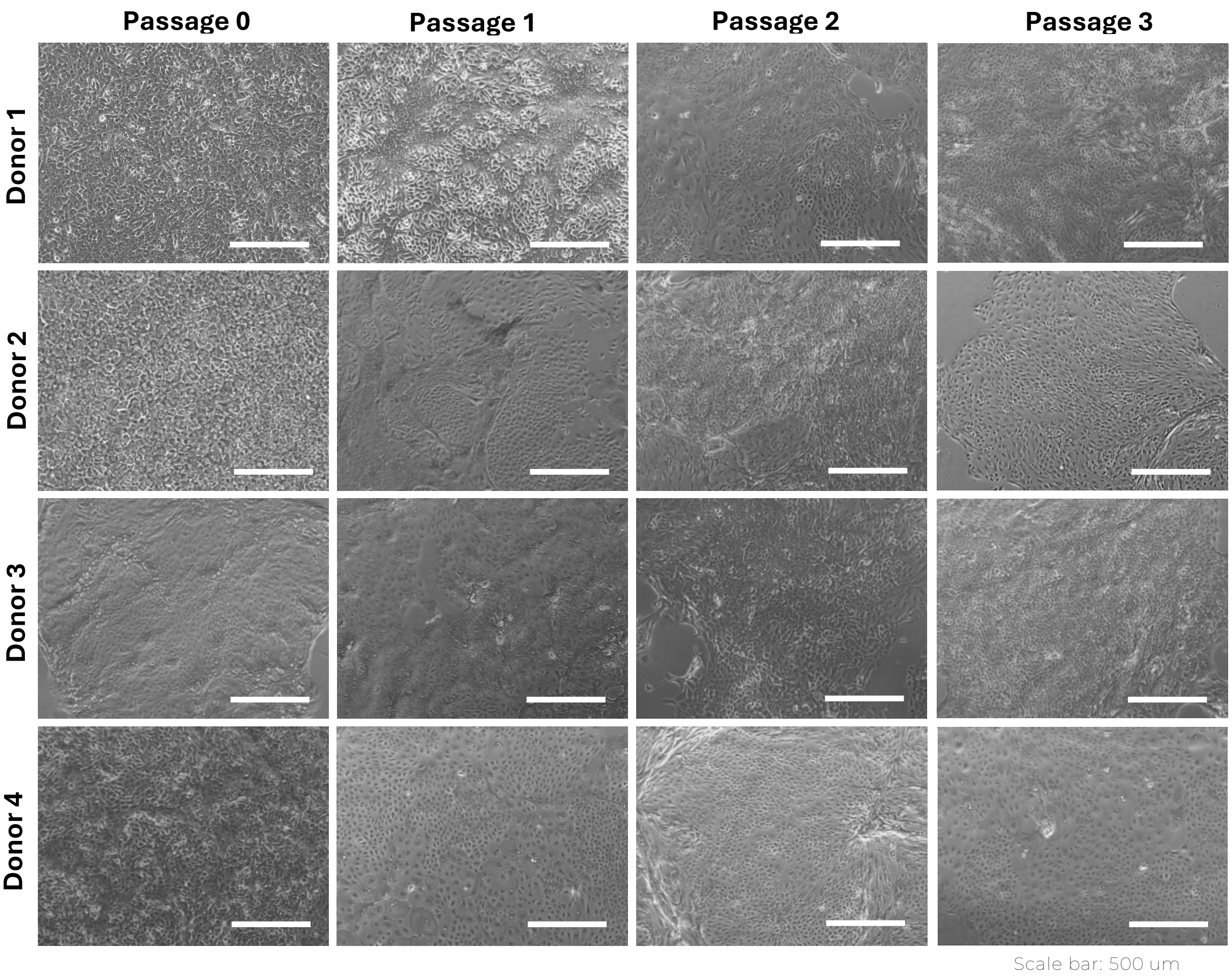

Figure S2: Milk cell expansion in 2D over serial weekly passaging

Expansion of milk derived cells from 4 different donors over serial weekly passaging. Brightfield images of cells at day 7 at 85-90 % confluency showing different colony types: refractive edges, cobble stone and stratified. Scale bar 500 µm.

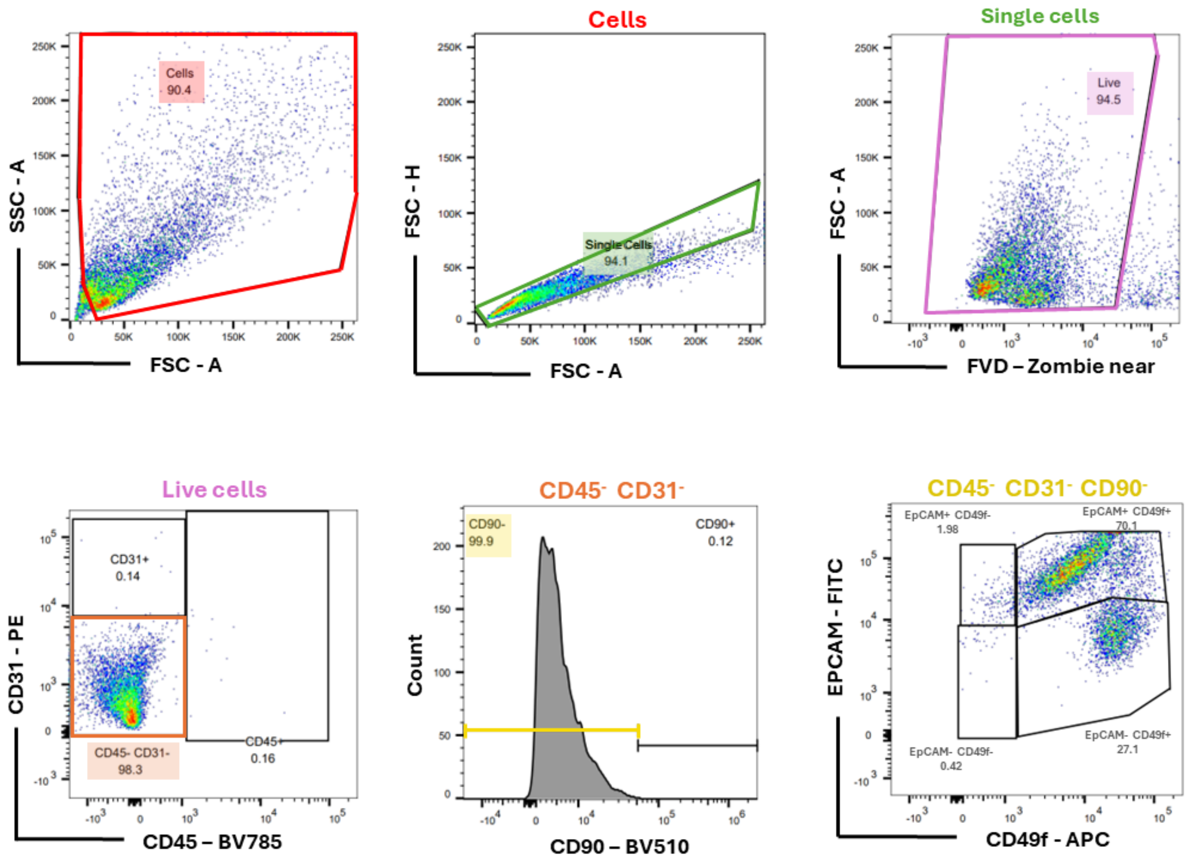

Figure S3: Flow Cytometry gating strategy for milk derived cells

Flow cytometric panel was designed using previously established protocols for breast tissue derived cells to identify mammary epithelial cells of basal and luminal epithelial phenotypes. Gates were color-coded to indicate parent and child populations. Red gate = all cells; green gate = single cells; purple gate = live cells; orange gate = CD45$^-$CD3$^-$ cells; yellow gate = Lin$^-$ (CD45$^-$ CD31$^-$ CD90$^-$) cells.

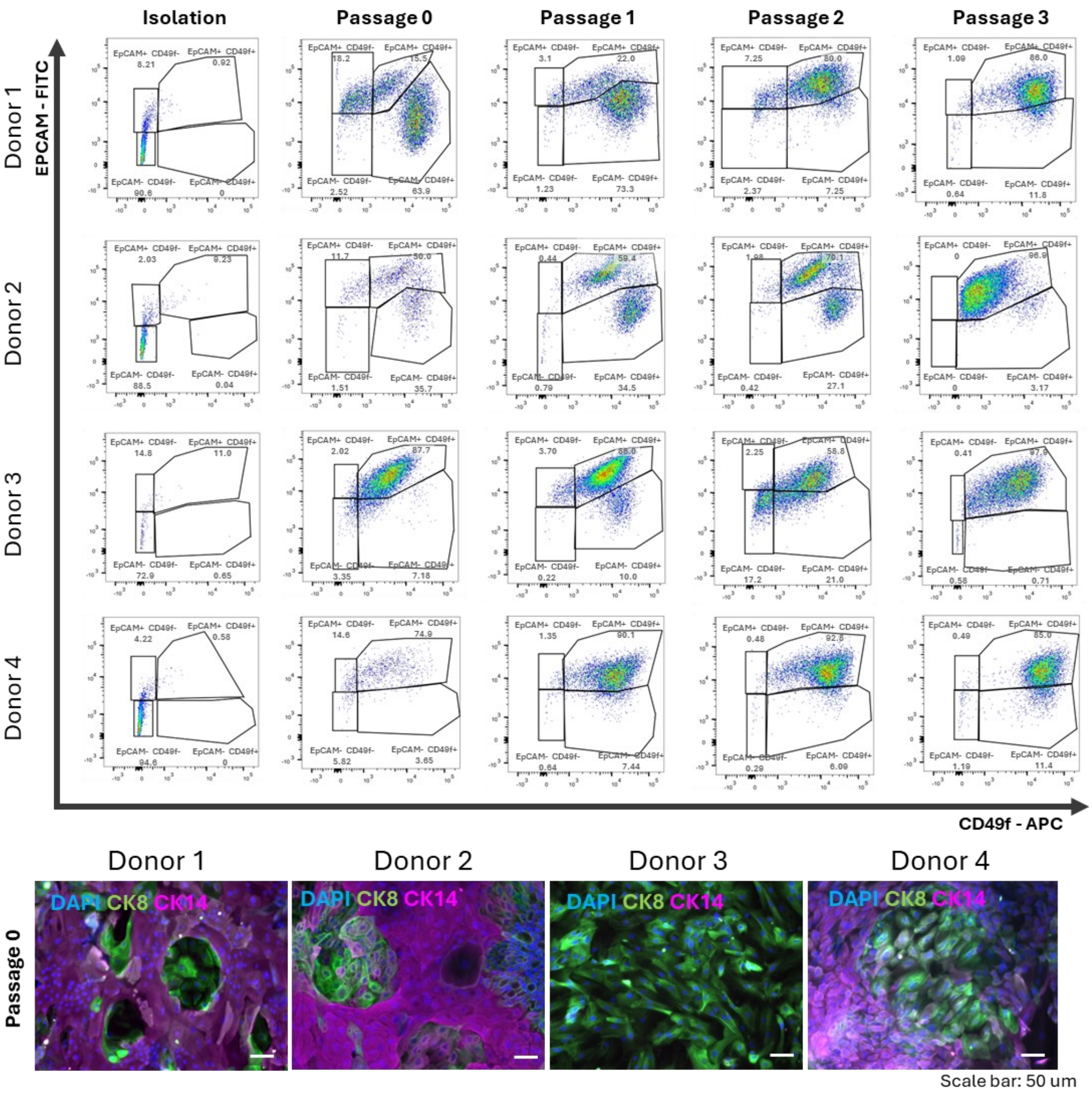

Figure S4: Mammary epithelial cell populations during isolation and over 3 passages analyzed by Flow cytometry

Top: Milk derived cells were cultured in vitro and split at 85-90 % confluency. Flow cytometric analysis showing mammary epithelial cell populations over passaging: The cells display mature luminal MEC (EPCAM+ CD49f-), luminal progenitor MEC (EPCAM+ CD49f+) and basal-like MEC phenotypes (EPCAM- CD49f+) (n= 4 donors).
Bottom: Immunofluorescent staining for CK8 and CK14 markers in P0 milk MEC from 4 donors.

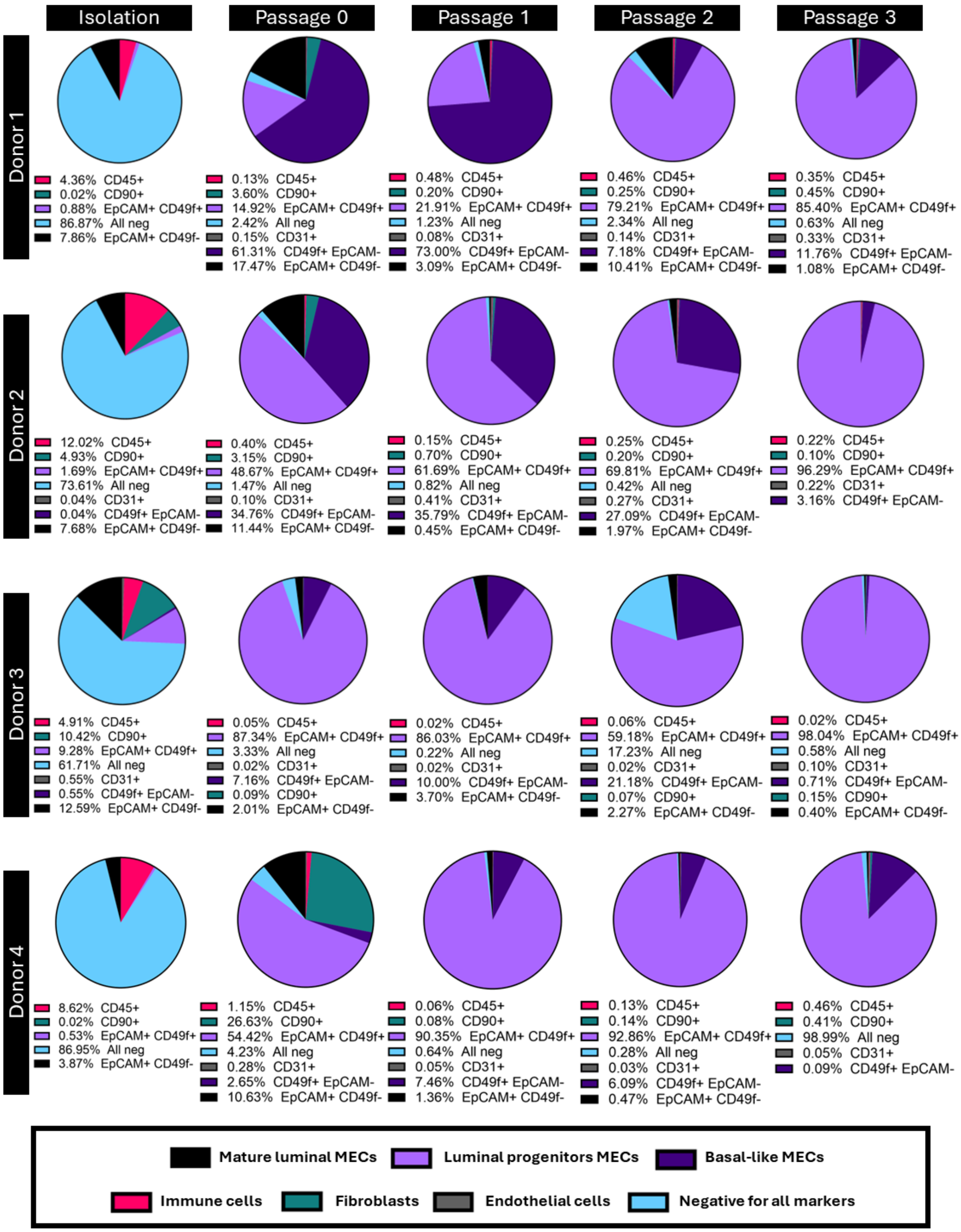

Figure S5: Overview of cell population dynamics during serial passaging.

Milk cells derived from 4 breastfeeding mothers were cultured *in vitro* and split at 85-80% confluency and analyzed by Flow Cytometry. Red corresponds to CD31$^-$ live cells expressing CD45 (immune cells), green are CD45$^-$ CD31$^-$ live cells expressing CD90 (fibroblasts and contractile type 1 basal cells), black corresponds CD45$^-$ live cells expressing CD31 (endothelial cells), cyan corresponds to cells that were negative for all the selected markers, dark purple are live CD45$^-$ CD31$^-$ EPCAM$^-$ but CD49f$^+$ cells (basal-like) and light purple corresponds to live cells CD31$^-$ CD45$^-$ expressing both EPCAM and CD49f (luminal).

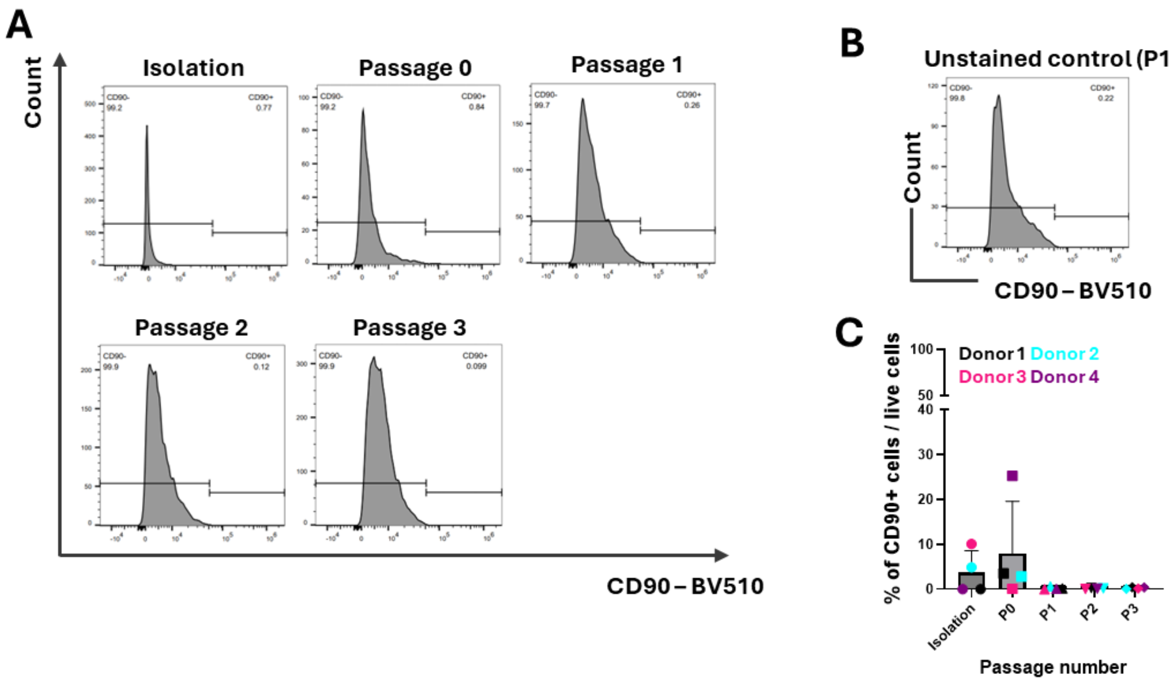

Figure S6: CD90 expression remained absent after P0 during 2D in vitro culture.

A) CD90 expression of $CD45^-$ / $CD31^-$ cells during isolation and 2D culture after 7 days of culture split at 85-90 % confluency. Y-axis: event count; X-axis: CD90 – BV510 signal. B) Unstained control of isolated mammary epithelial cells at passage 1. Y-axis: event count; X-axis: CD90 – BV510 signal. As cell autofluorescence increased during passaging, the CD90 gate was adjusted to account for the most autofluorescent cells, leading to a less stringent gating threshold, particularly in freshly isolated samples. Negative controls were recorded for each passage and donor, ensuring that gating was set appropriately. C) CD90 expression of $CD45^-$ / $CD31^-$ cells determined by Flow cytometric during isolation and 2D culture after 7 days of culture split at 85-90 % confluency (n=4 donors; mean ± SD, $p<0.05$)

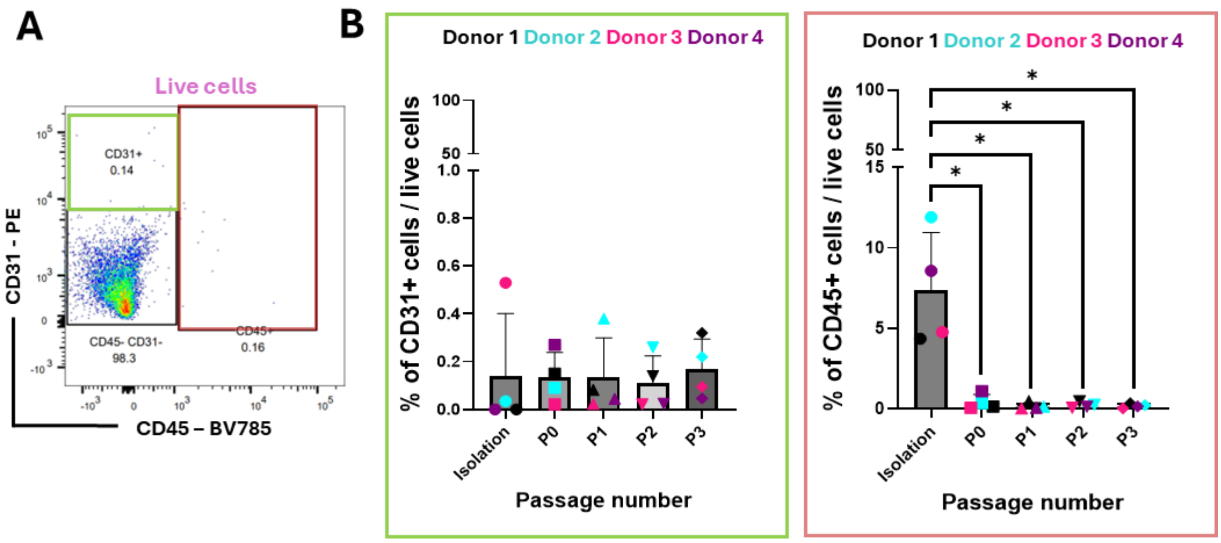

Figure S7: CD31+ and CD45+ live cells population from isolation and over 3 passages analyzed by Flow Cytometry

A) Flow cytometry gating strategy for identifying live cell populations of $CD31^+$ (endothelial) and $CD45^+$ (immune) cells; B) percentage of CD31 expressing cells (green gate) and CD45 expressing cells (orange gate) during isolation and over three passages. Percentages indicate the proportion of each gated population within the total live cell fraction. ($p<0.05$; n=4 donors; mean ± SD)

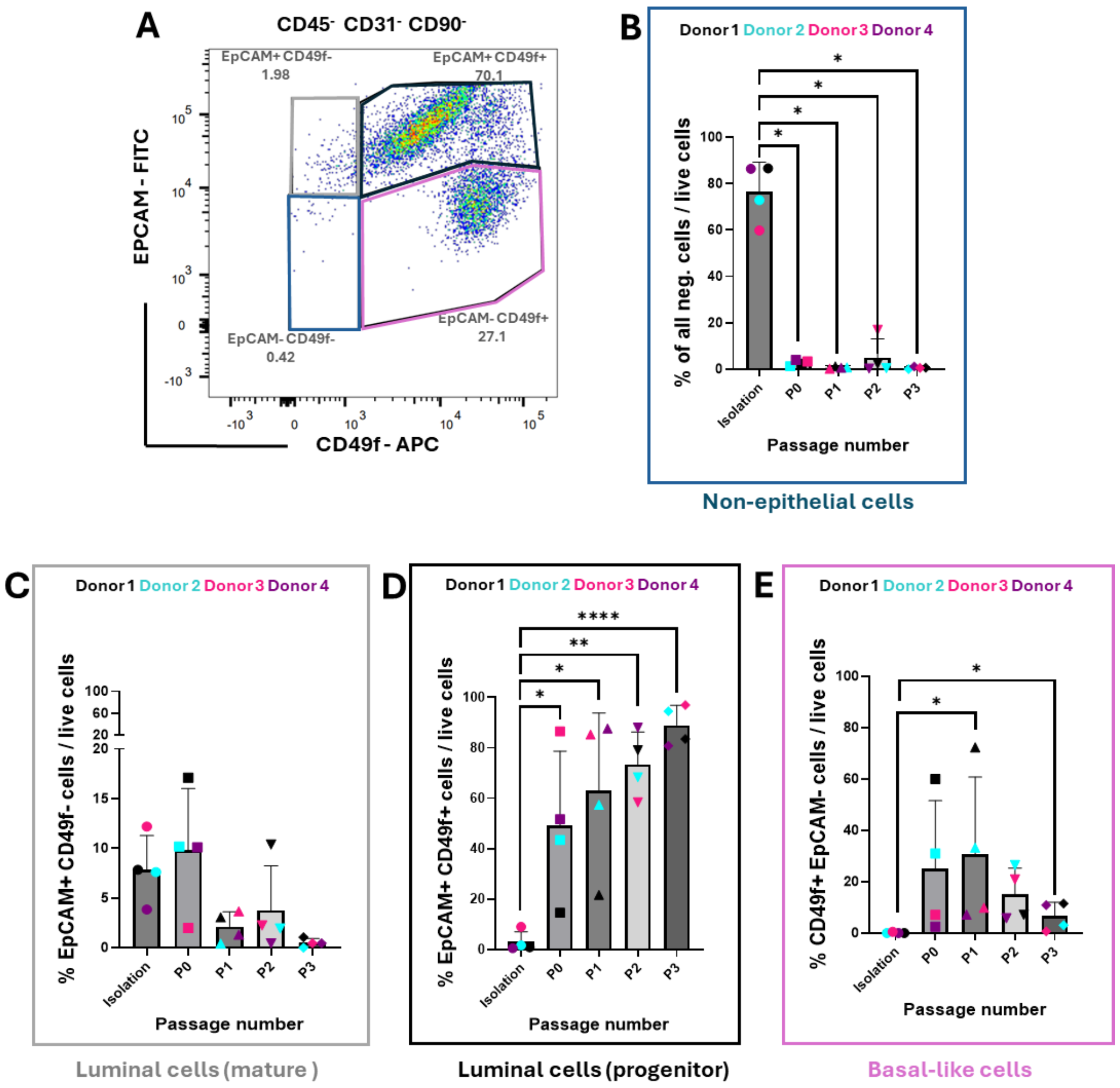

Figure S8: Overview of the CD45⁻ / CD31⁻ /CD90⁻ (lineage negative cells) cells identified by Flow Cytometry displaying epithelial phenotypes during isolation and culture over 3 passages.

A) Flow cytometry gating strategy for identifying live epithelial cell populations, defined as EPCAM$^+$ and/or CD49f$^+$ cells. Color-coded gates correspond to related panels B–E; B) Percentage of EPCAM and CD49f negative cells (non-epithelial phenotype); C-D) EPCAM positive and CD49 negative cells and double positive cells for EPCAM and CD49f corresponding to luminal mammary epithelial cells; E) EPCAM negative and CD49 positive cells (basal-like epithelial cells) (n=4 donors; mean ± SD; p<0.05 * - p<0.01 ** - p<0.001*** - p<0.0001****).

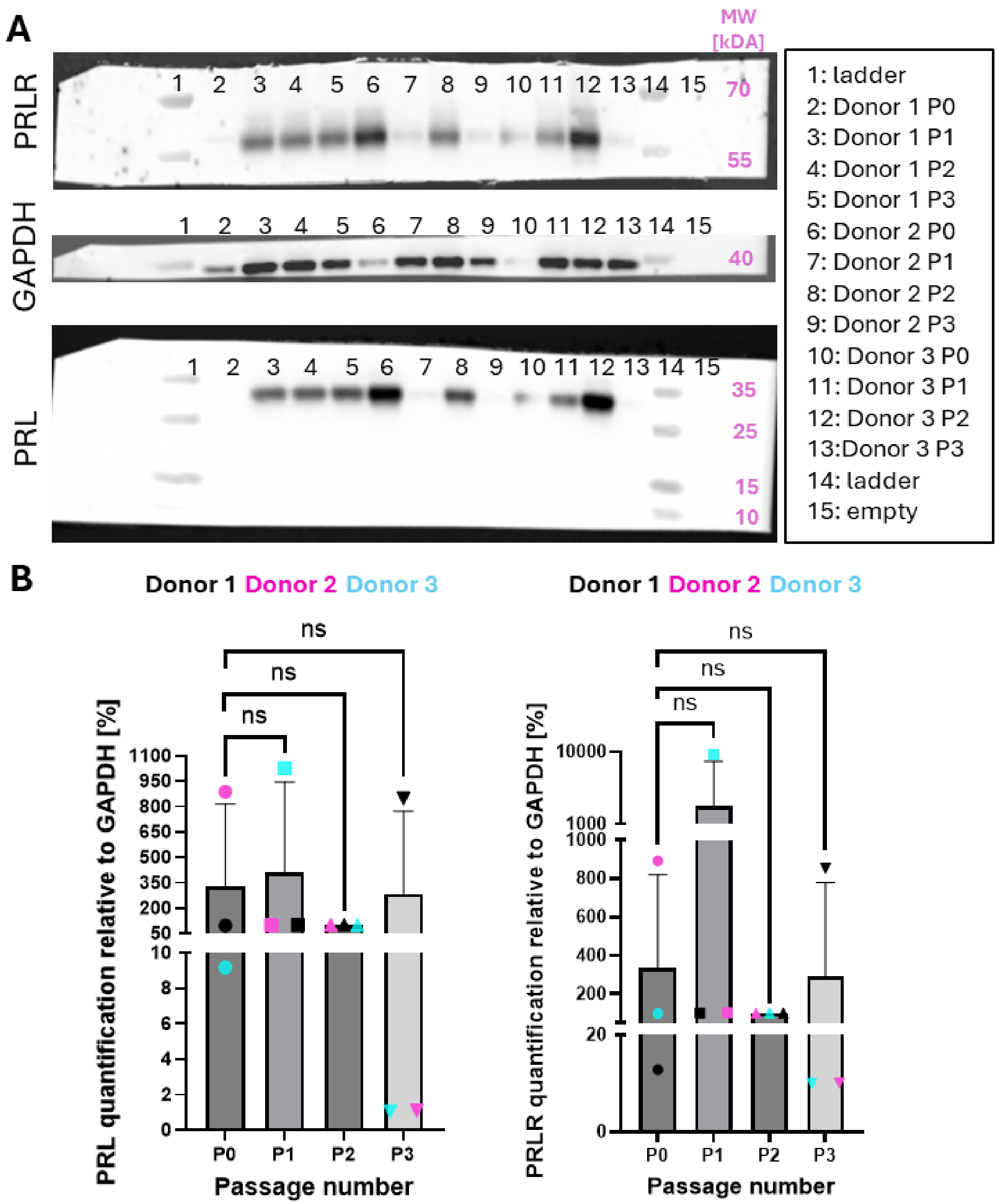

Figure S9: Western Blot

A) Full Western Blot from main figure 2. Numbers indicate the lanes, loaded samples are described in the black box. The molecular weight of the used ladder is indicated in purple. B) Western Blot quantification relative to GAPDH from 3 donors from passages P0 to P3, mean ± SD, $p<0.05$.

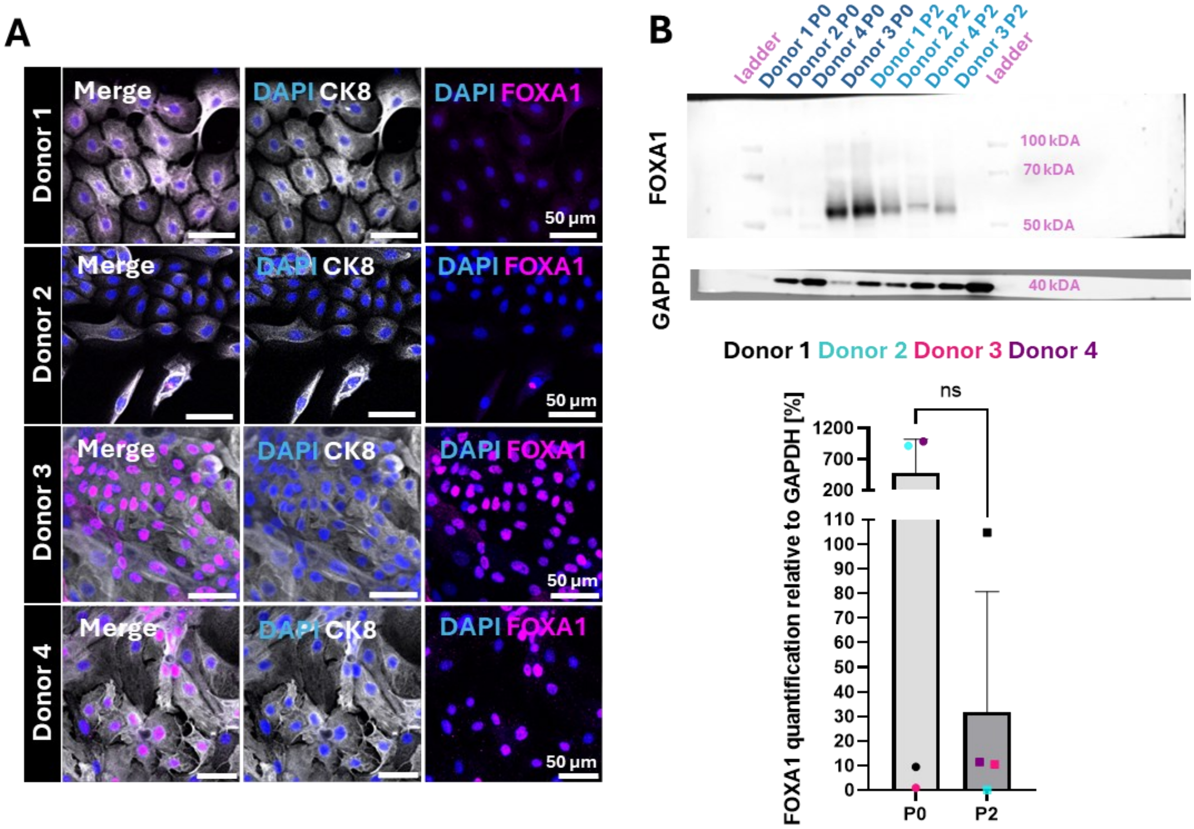

Figure S10: FOXA1 expression of mammary epithelial cells in 2D culture.

A) Confocal imaging of cells in 2D culture (P0, day 6) of 4 donors stained for FOXA1 (pink), CK8 (grey) and nuclei (blue). B) Western Blot of cells in culture from 4 different donors at P0 and P2 and corresponding quantification relative to GAPDH (n=4 donors, mean ± SD, $p<0.05$).

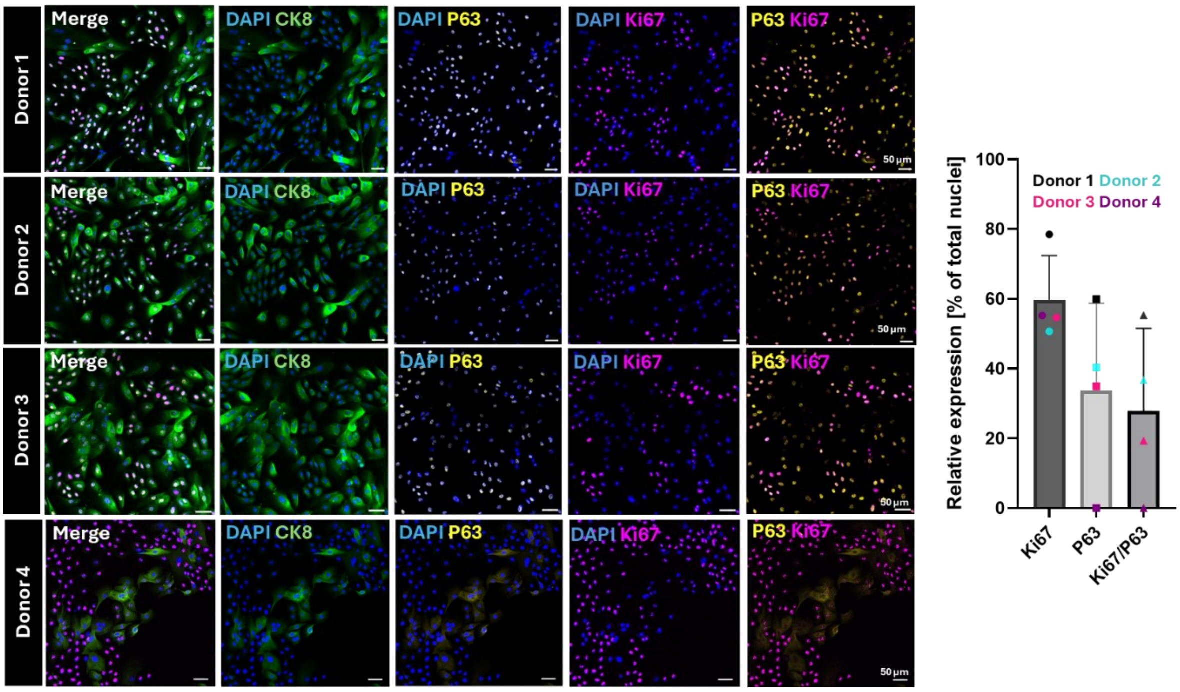

Figure S11: Immunofluorescent staining of cells in culture from 4 donors.

Selected regions of cytokeratin 8 (green), P63 (yellow) and Ki67 (pink) expression of cells in culture from 4 different donors (cells were at different passages, which may contribute to variability in the experimental outcomes), and imaging quantification performed in Image J using the "analyze particles" tool ($p<0.05$, n=4 donors; mean ± SD).

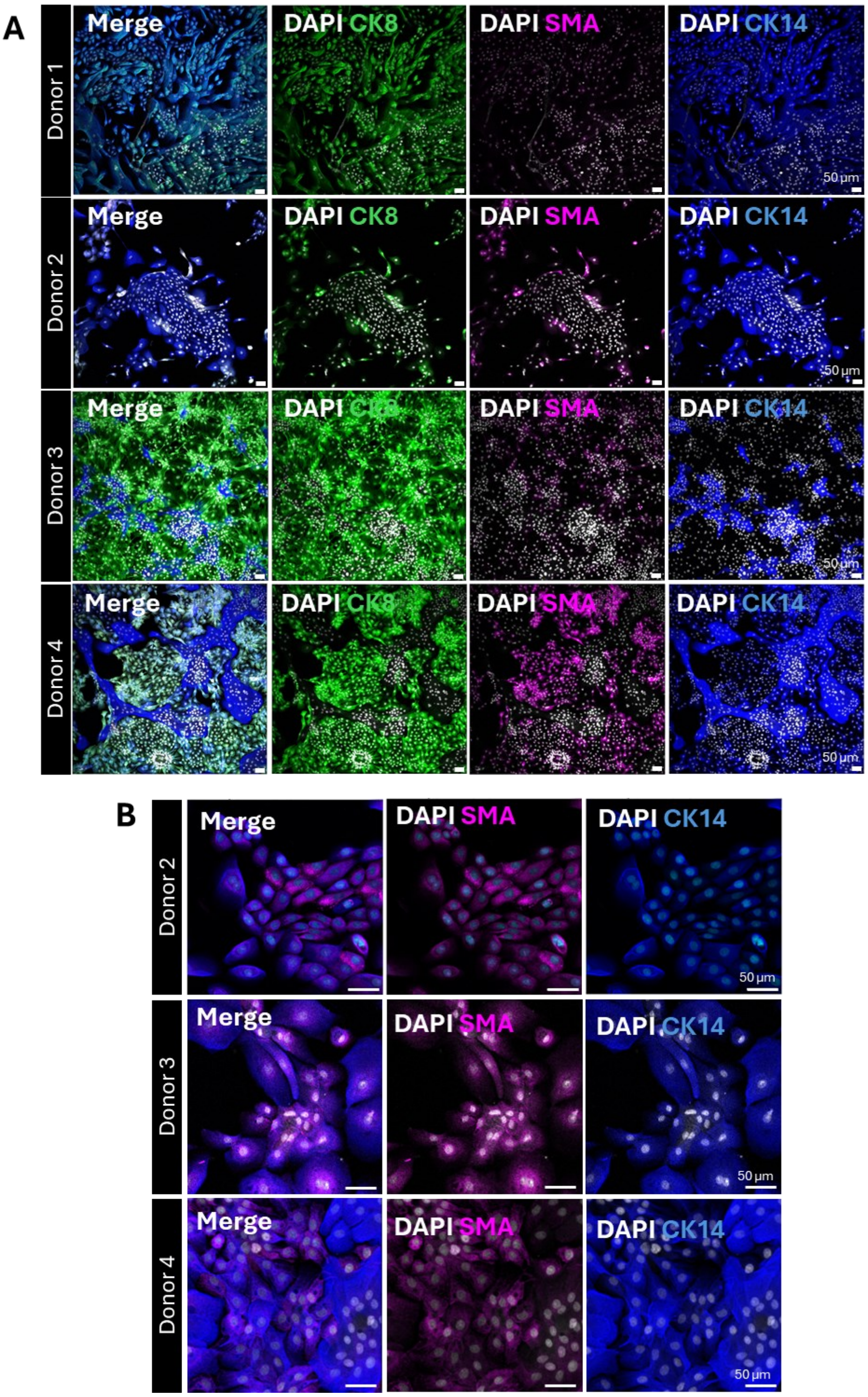

Figure S12: Immunofluorescence images of basal markers in milk-derived MECs (at P2) cultured in 2D.

A) Low-magnification immunofluorescence images showing Cytokeratin 8 (CK8, green), Smooth Muscle Actin (SMA, pink), and Cytokeratin 14 (CK14, blue) to identify luminal and basal cell populations. Nuclei are counterstained with DAPI (white). B) Higher-magnification images of selected regions displaying cells with possible co-expression of SMA and CK14, suggesting a basal phenotype. Further analysis is required to confirm this observation. Scale bars = 50 µm.

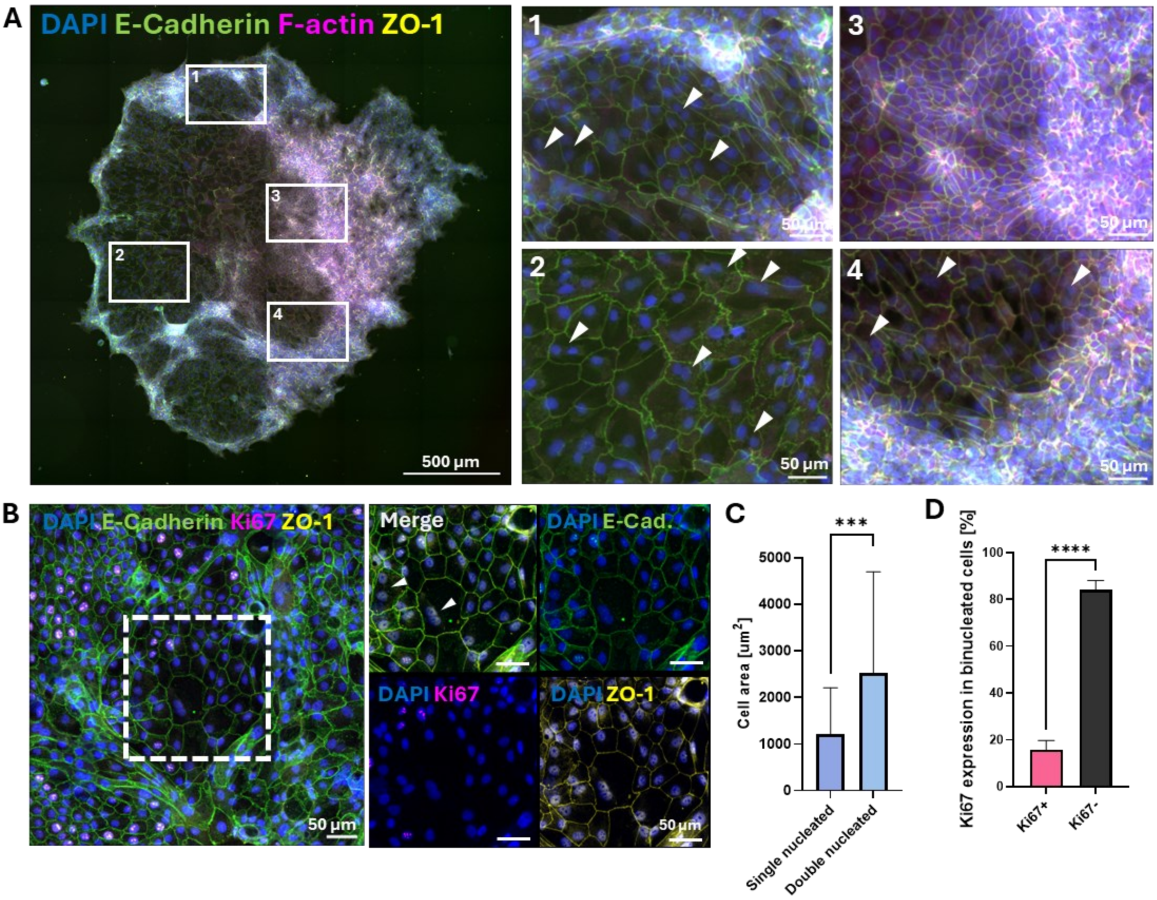

Figure S13: Tight junctions, proliferation and binucleation of milk MEC in culture.

A) Fluorescent microscopy images of representative milk MEC colony (P0) stained for DAPI (blue, nuclei), E-Cadherin (green, cell-cell junctions), F-actin (magenta, cytoskeleton), and ZO-1 (yellow, tight junctions). Left: Overview of the cell colony with selected regions of interest (white boxes). Right: Higher magnification images marked regions:1, 2 & 4: Areas with larger, examples of binucleated cells indicated with arrowheads; 3: Compact epithelial cell arrangement with mostly mononucleated cells. B) Representative immunofluorescence images showing the expression of epithelial junctional markers and proliferative activity in milk derived mammary epithelial cells at passage 2. Cells are stained for DAPI (nuclei, blue), E-cadherin (adherens junctions, green), Ki67 (proliferation marker, magenta), and ZO-1 (tight junctions, yellow). White arrowheads indicate examples of binucleated cells. Scale bars: 50 µm. C) Cell area quantification from single and double nucleated cells (n=3 independent cultures; mean ± SD). D) Quantification of Ki67 expression in binucleated cells. Among these cells, 80% are Ki67-negative, suggesting quiescence (not in G2/M), while 20% are Ki67-positive, indicating active proliferation. (n=3 independent cultures; mean ± SD, $p<0.05$ * - $p<0.01$ ** - $p<0.001$*** - $p<0.0001$****)

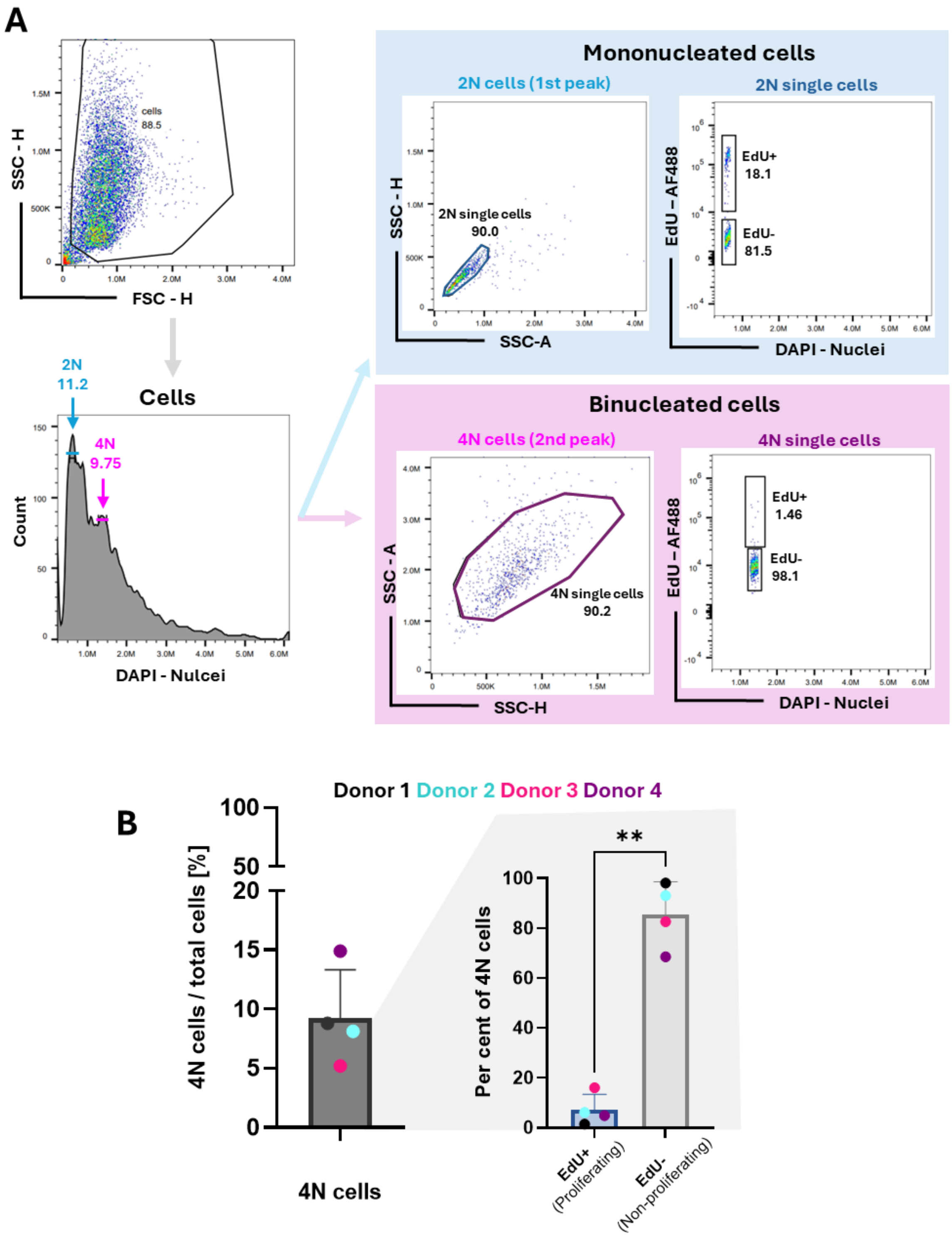

Figure S14: Flow cytometric analysis of binucleated cells and cell cycle assessment using EdU.

A) Cells in culture (P2) were stained with EdU (to mark proliferation i.e. cells actively synthesizing DNA) and DAPI for the nuclei.
B) Quantification of 4N (binucleated) cells was conducted across four biological replicates. Approximately 9% of the total cell population were binucleated, with ~80% of these cells being non-proliferative. Data are presented as mean ± SD, n = 4 donors and $p < 0.01$.

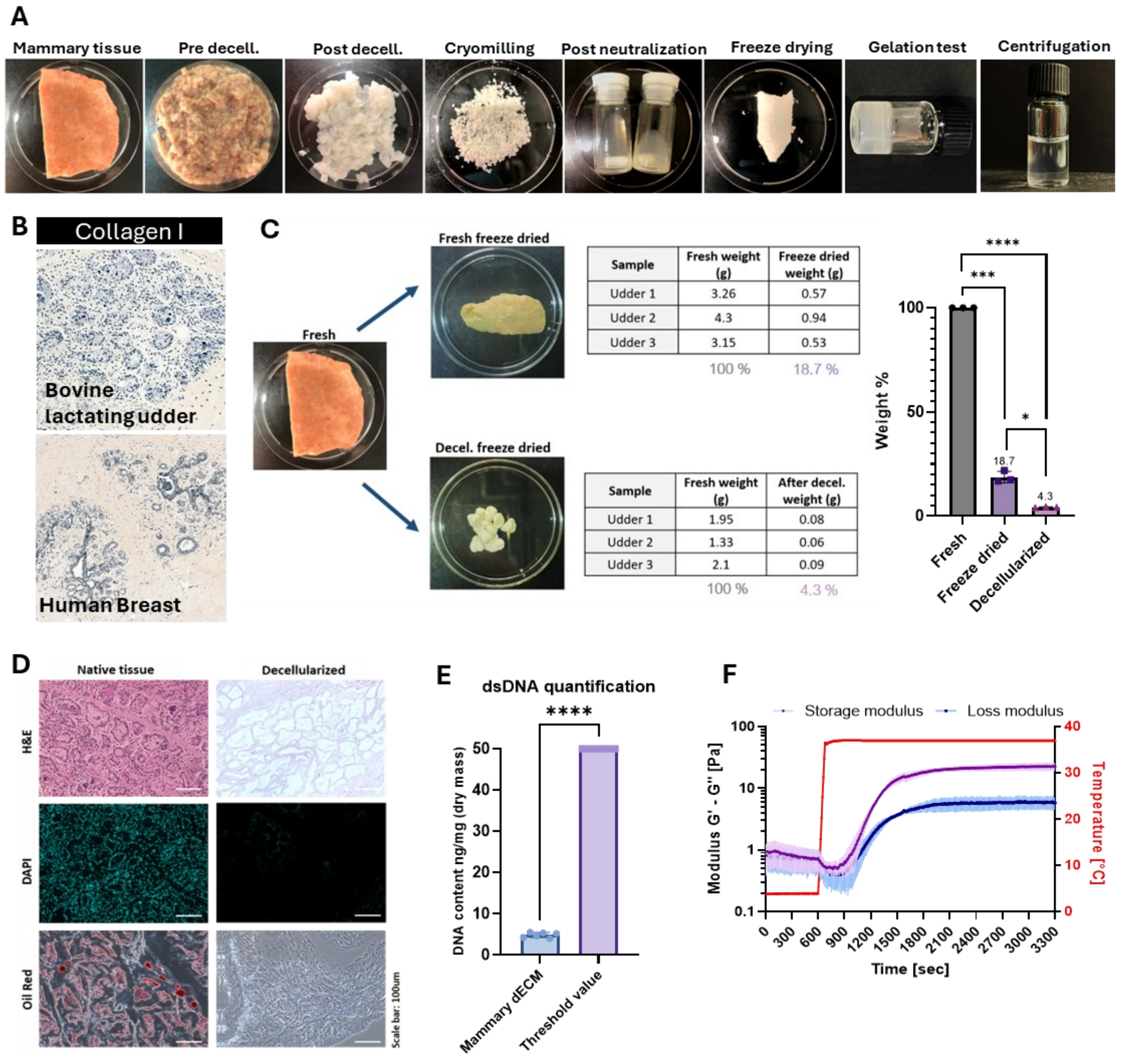

Figure S15: Tissue decellularization

A) Decellularization process: Bovine mammary tissue was (i) harvested, (ii) decellularized, (iii) freeze dried and cryomilled, (iv) digested and neutralized, and (v) freeze drying again for long term storage. Weight percent of freeze dried and freeze dried and decellularized tissue vs. fresh bovine mammary tissue (n= 3 biological replicates). B) Col-I stainings of histological sections of bovine and human mammary tissue. C) Weight before (fresh tissue) vs. after decellularization and freeze drying. (n=3 biological replicates, mean ± SD). D) Histological comparison of tissue structure (H&E), of cell (DAPI) and lipid (Oil RED) content between the native vs. decellularized bovine mammary tissue. E) Double stranded DNA quantification of the decellularized mammary tissue vs. the published threshold value (50 ng/ml) indicating a successful cell removal (n=6 biological replicates, mean ± SD). F) Rheological profile of the centrifuged $dECM_{mam}$ batches resuspended at 40mg/ml in cold PBS with a significant increase ($p < 0.0001$) in both the storage and loss modulus following the temperature-induced gelation, compared to the material at 4°C. p<0.05 * - p<0.01 ** - p<0.001*** - p<0.0001****

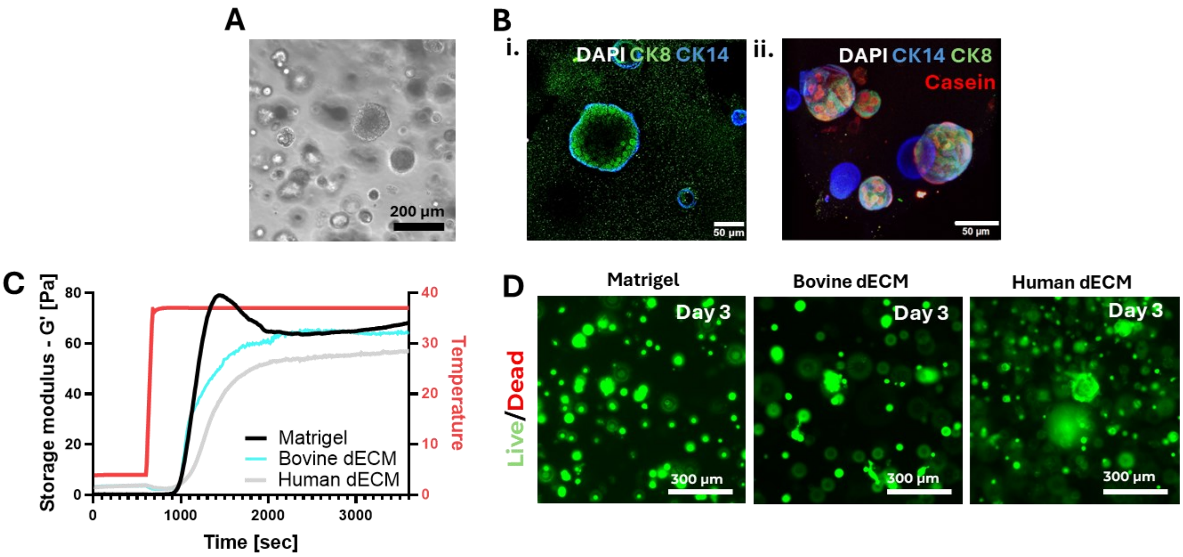

Figure S16: Milk-derived MECs cultured in 3D environments composed of Matrigel and mammary dECM.

A) Representative bright field imaging of milk derived MECs grown in Matrigel (day 9) with B) corresponding immunofluorescent images: i) image slice of cytokeratin 8 (CK8, green) and Cytokeratin 14 (CK14, blue) staining and ii) 3D reconstructed z-stack of CK14 (blue), CK8 (green) and beta-casein (red) imaging; scale bars: 50 µm. C) Rheological measurements of bovine (20 mg/ml) and human (20 mg/ml) mammary dECM (uncentrifuged) compared to Matrigel. Bovine and human mammary dECM achieve similar sigmoidal gelation curves compared to Matrigel. D) Live/dead staining of milk MEC cultured in thermally gelled Matrigel, bovine mammary dECM and human mammary dECM on day 3.

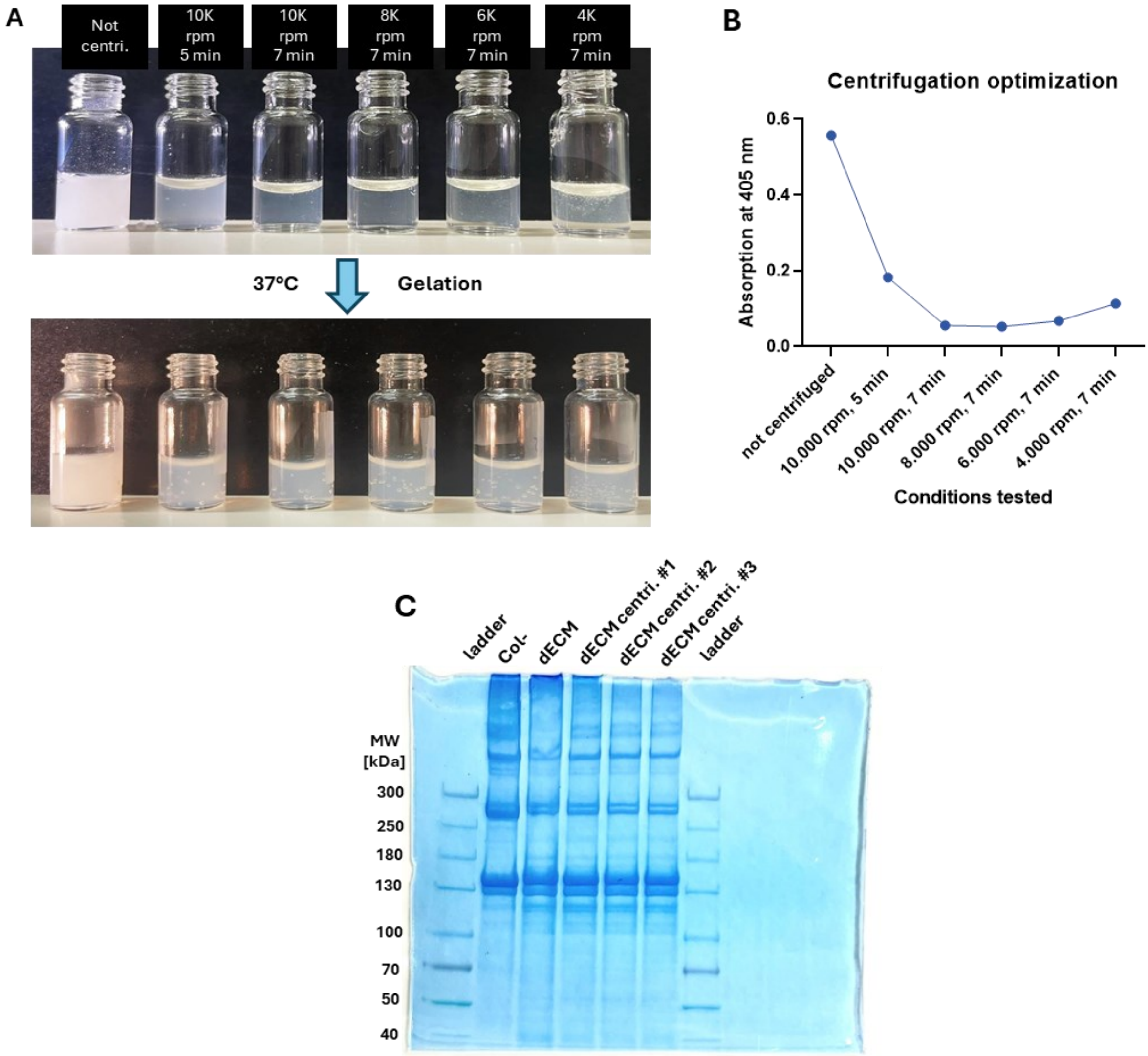

Figure S17: Optimization of optical properties by centrifugation

A) $dECM_{mam}$ (40mg/ml) centrifuged at 4°C at different speeds and time, maintaining its self-gelling capacities when placed at 37°C. B) Measurement of the absorption of the $dECM_{mam}$ centrifuged under different conditions at 4°C in the liquid state. C) Biochemical composition of the $dECM_{mam}$ gels via SDS Page compared to Col-I.

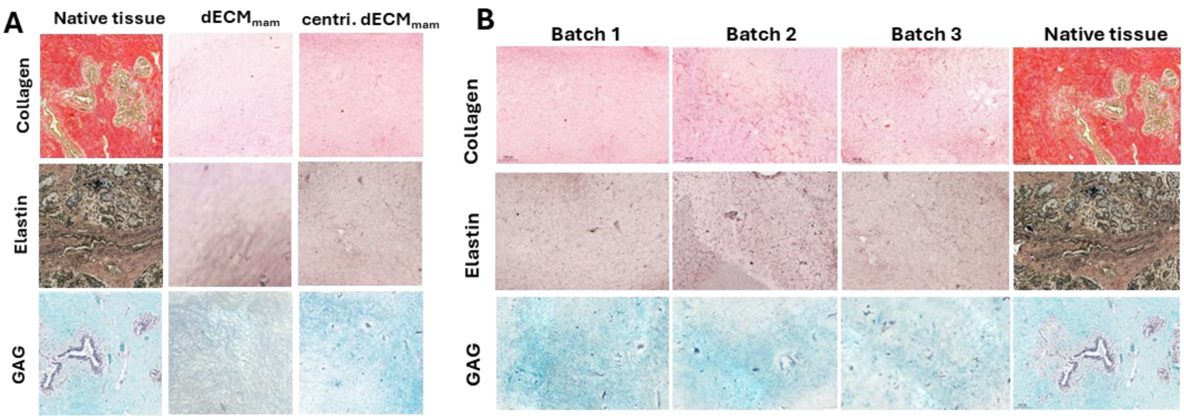

Figure S18: Histological identification of main functional components of the ECM

A) Histological sections of fixed native tissue and (centrifuged) $dECM_{mam}$ gels stained with Picrosirius Red, Verhoeff's and Safranin-O for collagen, elastin and glycosaminoglycans, respectively, showing the presence of these functional components in the native tissue, as well as in the $dECM_{mam}$ gels pre and post centrifugation resuspended at 40 mg/ml in PBS, B) and comparison of the three centrifuged $dECM_{mam}$ batches resuspended at 40 mg/ml to the native tissue.

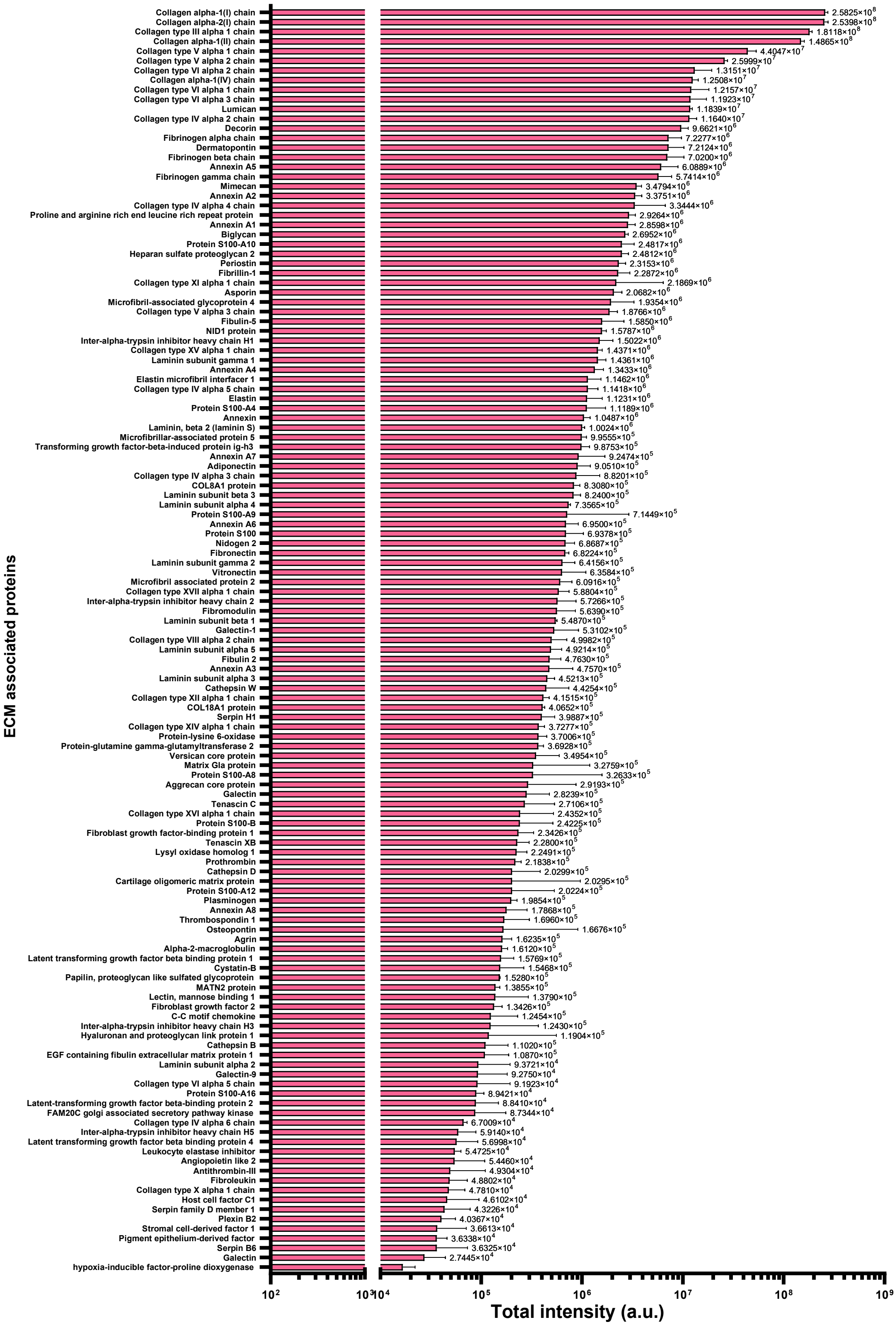

Figure S19: Relative abundance of all 132 ECM proteins identified by mass spectrometry.

A

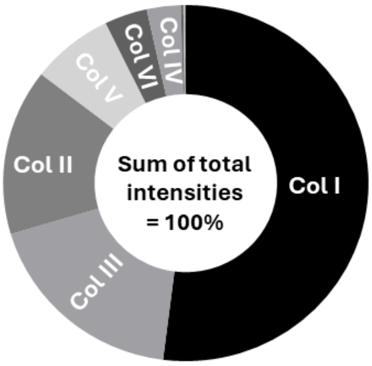

Collagens	Distribution [%]
Col I	51.99
Col III	18.39
Col II	15.09
Col V	7.30
Col VI	3.79
Col IV	3.00
Col XI	0.22
Col XVII	0.06
Col VIII	0.05
Col XII	0.04
Col XIV	0.04
Col XVI	0.02
Total	100.00

B

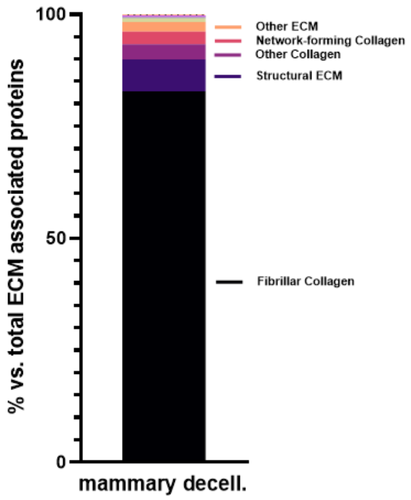

ECM associated proteins	Distribution [%]
Fibrillar Collagen	82.82
Structural ECM	7.13
Other Collagen	3.37
Network-forming Collagen	2.80
Other ECM	2.17
Signalling	0.79
Protease Inhibitor	0.30
Matricellular	0.27
Proteases	0.11
FACIT Collagen	0.09
ECM regulators (ECM enzymes)	0.09
Membrane Collagen	0.05
ECM PTM Modifier	0.01
Total	100

C

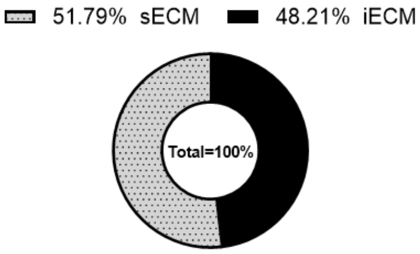

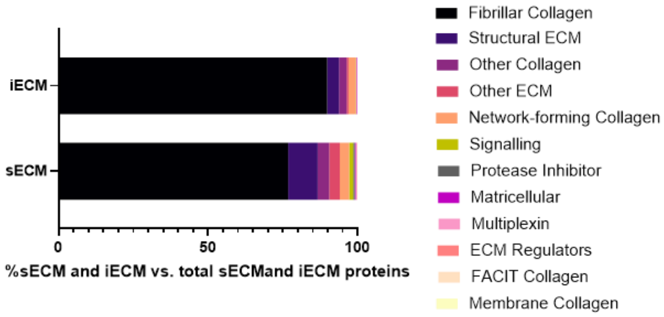

Figure S20: Proteomic analysis

A) Abundance of ECM-associated proteins based on DAVID gene ontology functional groups. Fibrillar collagens are the most abundant group found in the bovine mammary decellularized matrix. B) Percentage of proteins identified within the sECM, and iECM fractions of bovine decellularized mammary gland according to the DAVID gene ontology functional group classification.

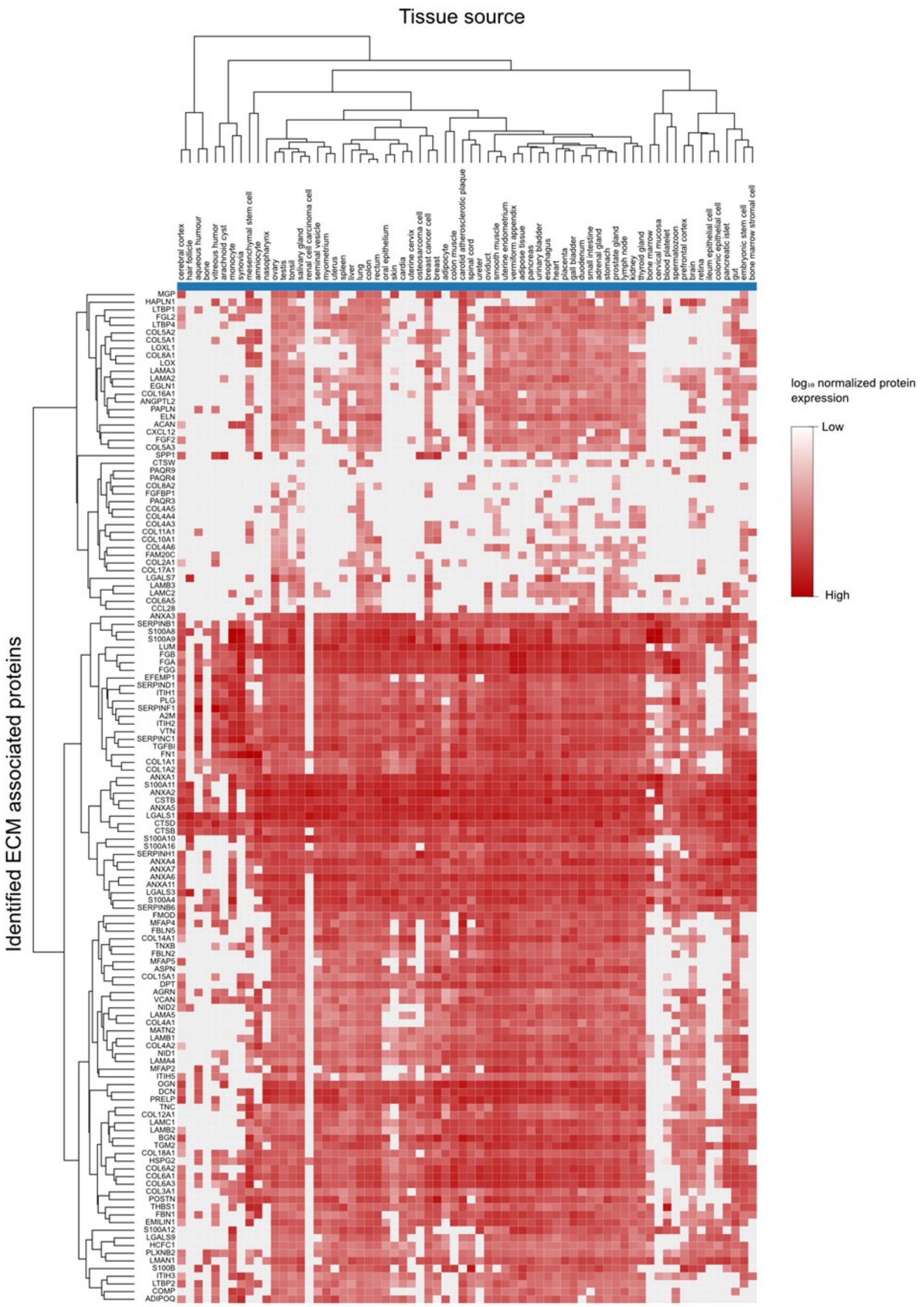

Figure S21: Similarities between the extracted bovine mammary ECM and human tissues.

Heatmap of the hierarchical clustering analysis of the 131 ECM associated proteins identified in the decellularized bovine tissue and compared to the human tissue data from a draft map of the human proteome made publicly available proteomicsdb.org. The analysis was restricted to relevant tissues for regenerative and tissue engineering applications. The 131 identified proteins were predominantly found in ectoderm derived tissue (e.g. epithelial and nerve tissues).

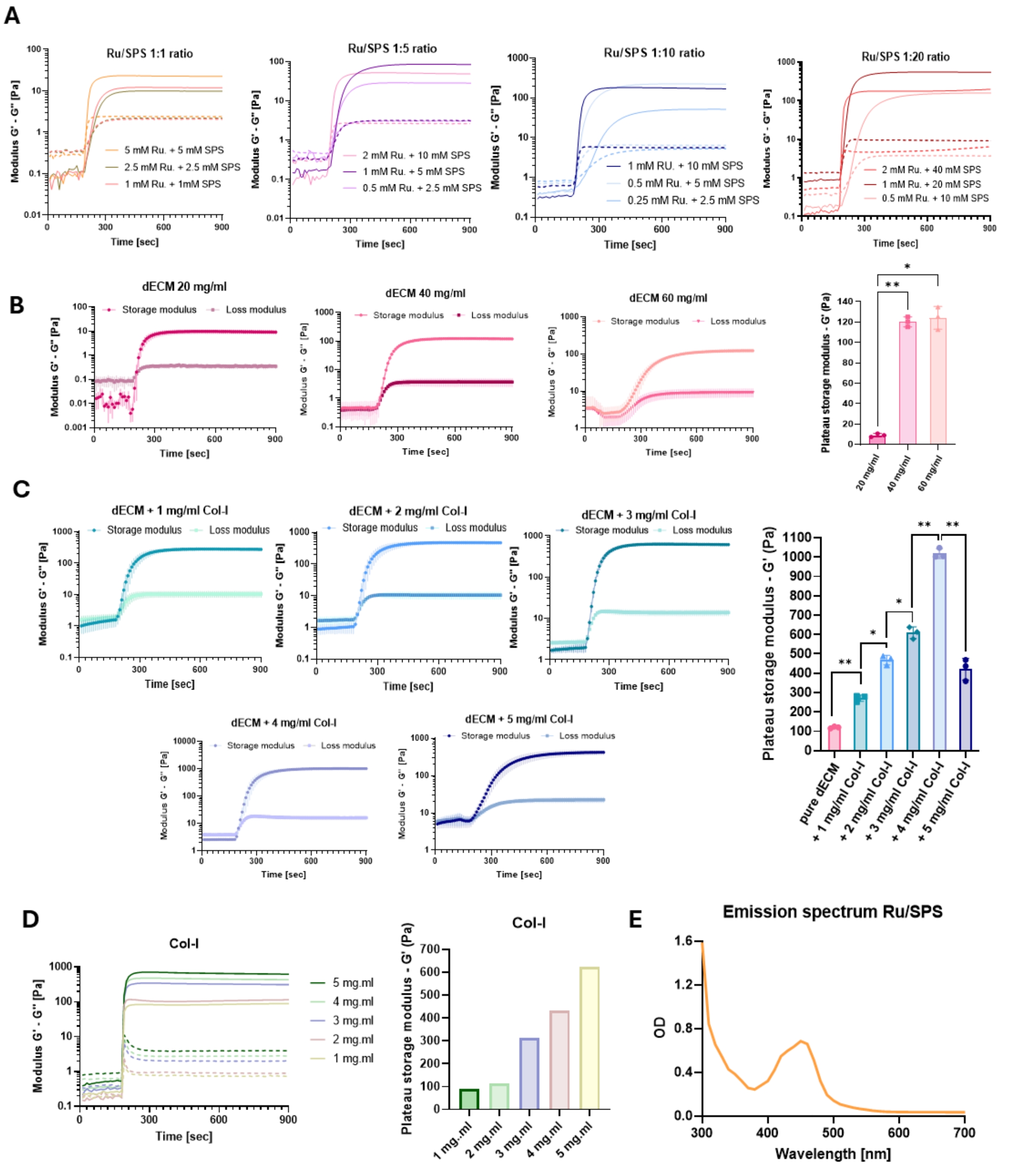

Figure S22: Optimization of resin composition and photoinitiator ratios.

A) Photorheological profile of the $dECM_{mam}$ (40mg/ml) + Col-I (4mg/ml) resin with different Ru/SPS concentrations and ratios. B) Photorheological profile of different 3 $dECM_{mam}$ concentrations with 0.5mM RU/5mM SPS (n= 3 $dECM_{mam}$ batches, mean ± SD). C) To make the 3 $dECM_{mam}$ resin stiffer Col-I was added to the resin in different concentrations (1 to 5 mg/ml); 0.5mM Ru/ 5mM SPS) (n= 3 $dECM_{mam}$ batches, mean ± SD). D) Photorheological characterization of Col-I at different concentrations (1 to 5 mg/ml); 0.5mM Ru / 5mM SPS. E) Emission spectrum of the used Ru/SPS photoinitiator system with the absorption maximum at 450 nm. $p<0.05$ * - $p<0.01$ ** - $p<0.001$*** - $p<0.0001$****

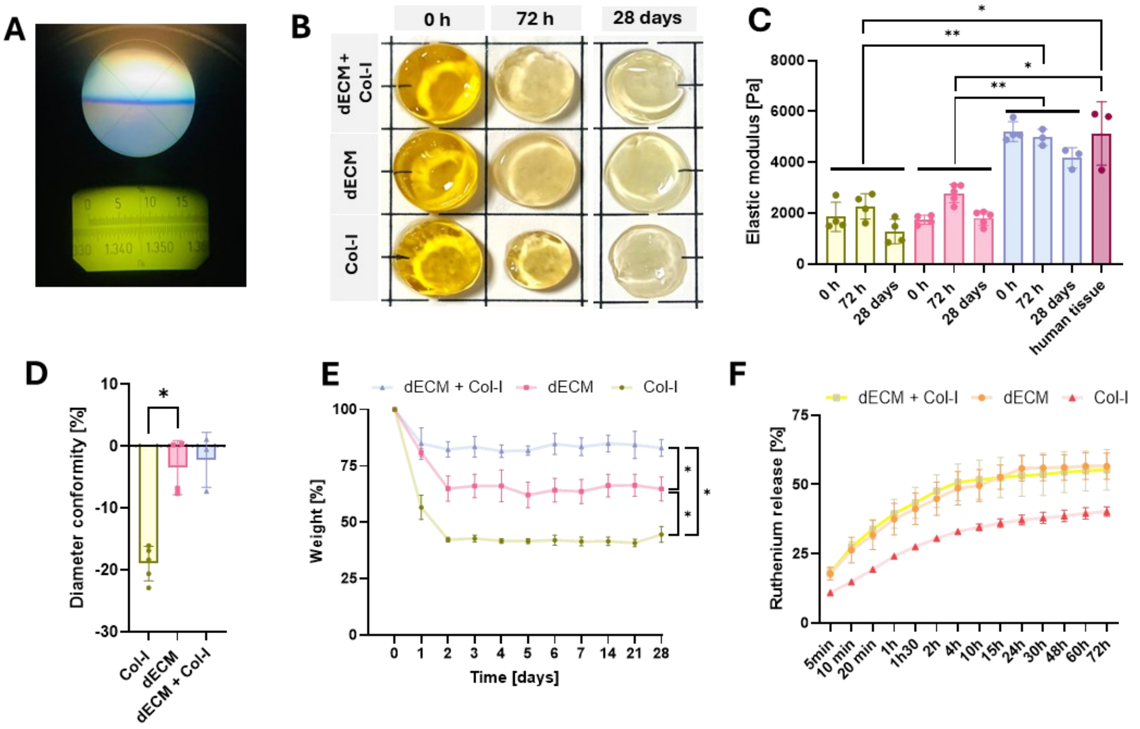

Figure S23: dECM/Col-I photoresin for mammary tissue engineering.
A) Refractive index measurement of the $dECM_{mam}$ / Col-I resin of 1.345. B) Assessment of the stability of volumetrically printed (VP) disks, over the time span of one month at RT in PBS on a shaker 150 rpm. C) Elastic moduli of the Col-I (4mg/ml), $dECM_{mam}$ (40 mg/ml) and $dECM_{mam}$ + Col-I (40 mg/ml + 4 mg/ml) VP printed disks post printing, after 3 days, and after one month at RT, in PBS on the shaker 150 rpm. D) The $dECM_{mam}$/Col-I was most stable as also shown by the analysis of the disk diameters. E) Measurement of the weight VP disks over one month. F) Analysis of the RU/SPS release from the VP printed disk by photometric analysis of the PBS solution. For C-F data are presented as mean ± SD, n=4 technical replicates, $p<0.05$* - $p<0.01$** - $p<0.001$*** - $p<0.0001$****

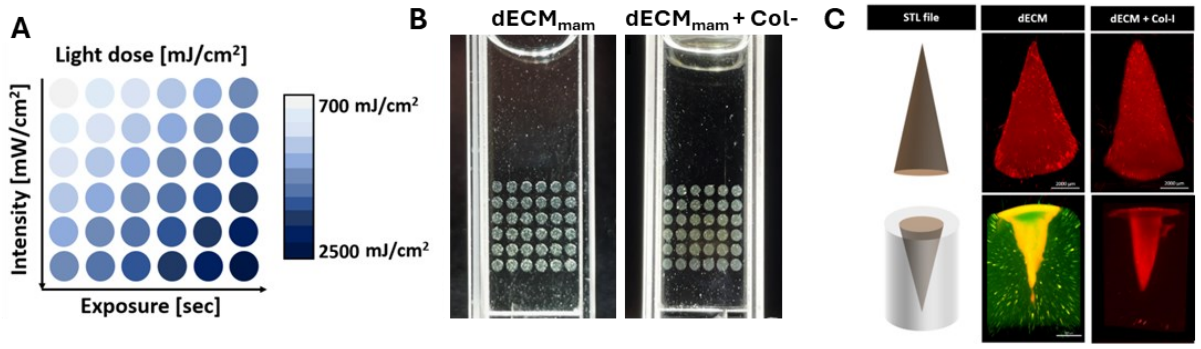

Figure S24: Volumetric printing of dECM and dECM/Col-I

A) Dose test set-up from 700 to 2500 mJ/cm2 to determine the printing window. B) $dECM_{mam}$ and $dECM_{mam}$/Col-I were mixed with 0.5mM RU/5mM SPS as photo initiator, and the doses test was projected in 1mm glass cuvettes containing the resins. C) Positive and negative resolution tests of the $dECM_{mam}$ and $dECM_{mam}$/Col-I printed with 2000 mJ/cm^2 and 1200 mJ/cm^2 respectively.

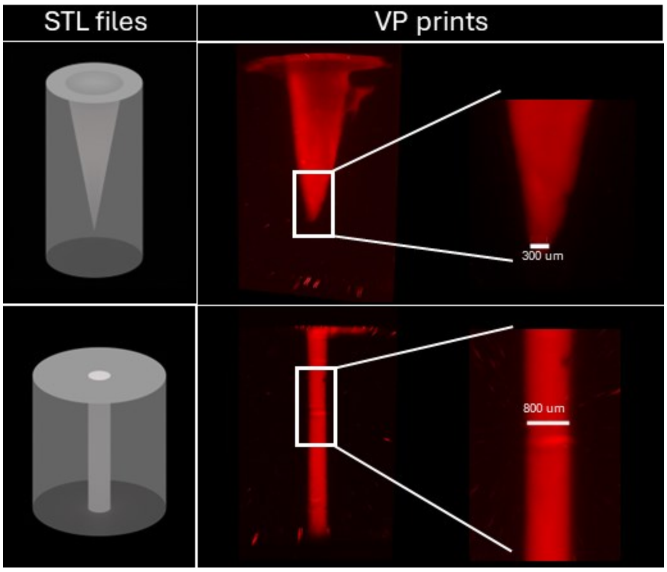

Figure S25: Volumetric printing: STL files VS. printed constructs

Constructs printed with $dECM_{mam}$/Col-I (40 mg/ml + 4mg/ml) with 0.5 mM RU and 5mM SPS; 1250 mJ/cm^2, printing time: 120 seconds

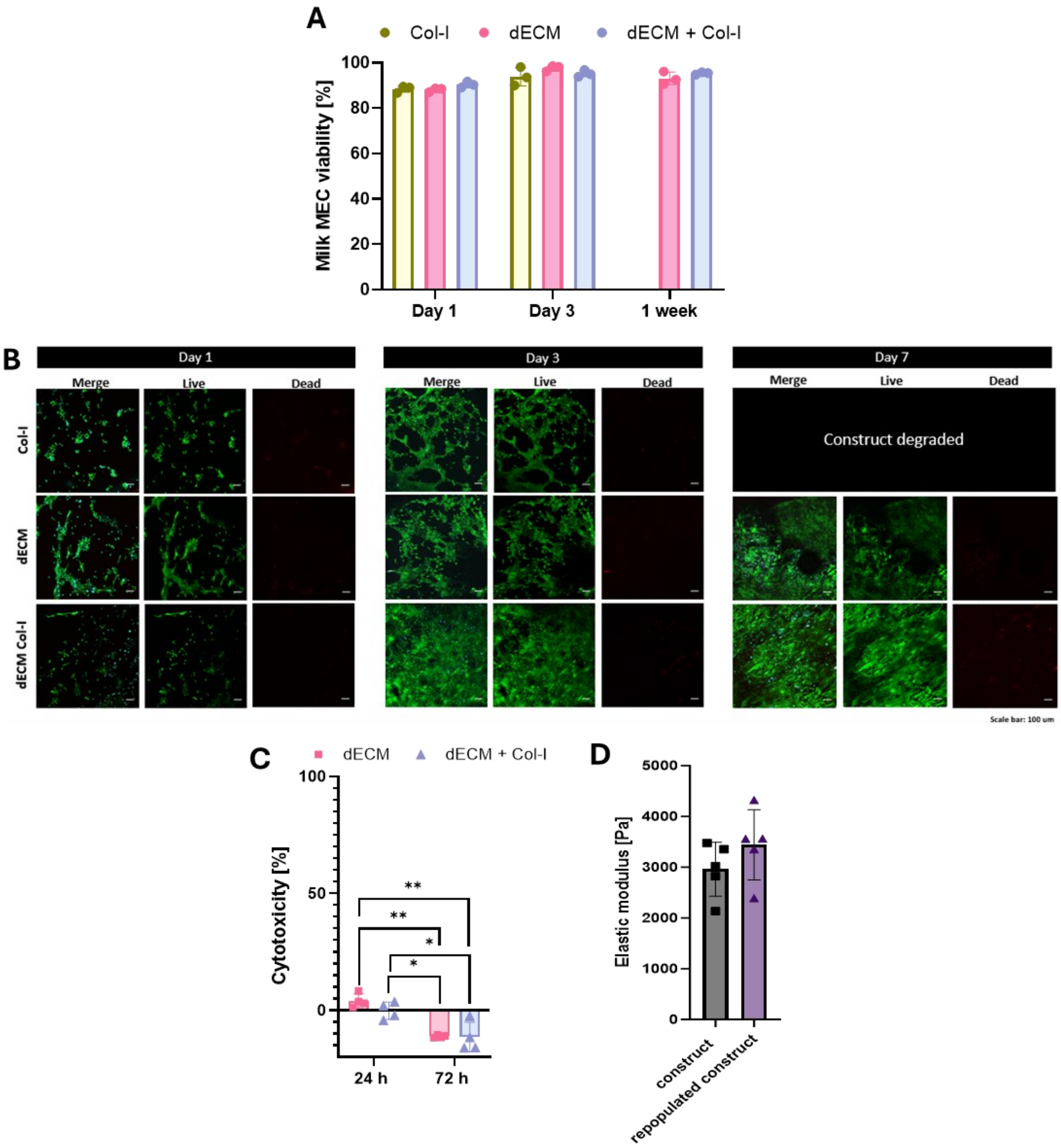

Figure S26: Biocompatibility of milk mammary epithelial cells (milk MEC) with printed dECM + Col-I constructs.

A) Milk MEC viability seeded on printed $dECM_{mam}$ and $dECM_{mam}$/Col-I constructs. Viability was assessed with Live/dead staining at day1, 3 and 7 days post seeding (n=3 biological replicates, mean ± SD). B) Confocal images of Calcein (green; live) and propidium iodide (red; dead) stained cells seeded on $dECM_{mam}$ and $dECM_{mam}$/Col-I constructs at 1, 3 and 7 days post seeding. C) Bar plot representing the cell cytotoxicity in percent for the milk MEC cultured on the $dECM_{mam}$ and $dECM_{mam}$/Col-I constructs for 24 and 72 hours. Cytotoxicity was assessed using the lactate dehydrogenase assay (n= 4 biological replicates, mean ± SD). D) Elastic moduli of $dECM_{mam}$/Col-I constructs with and without cells seeded after 1 week of culture in 37°C (seeding density: 10.000 cells/cm^2) (n= 5 biological replicates, mean ± SD) $p<0.05$* - $p<0.01$** - $p<0.001$*** - $p<0.0001$****

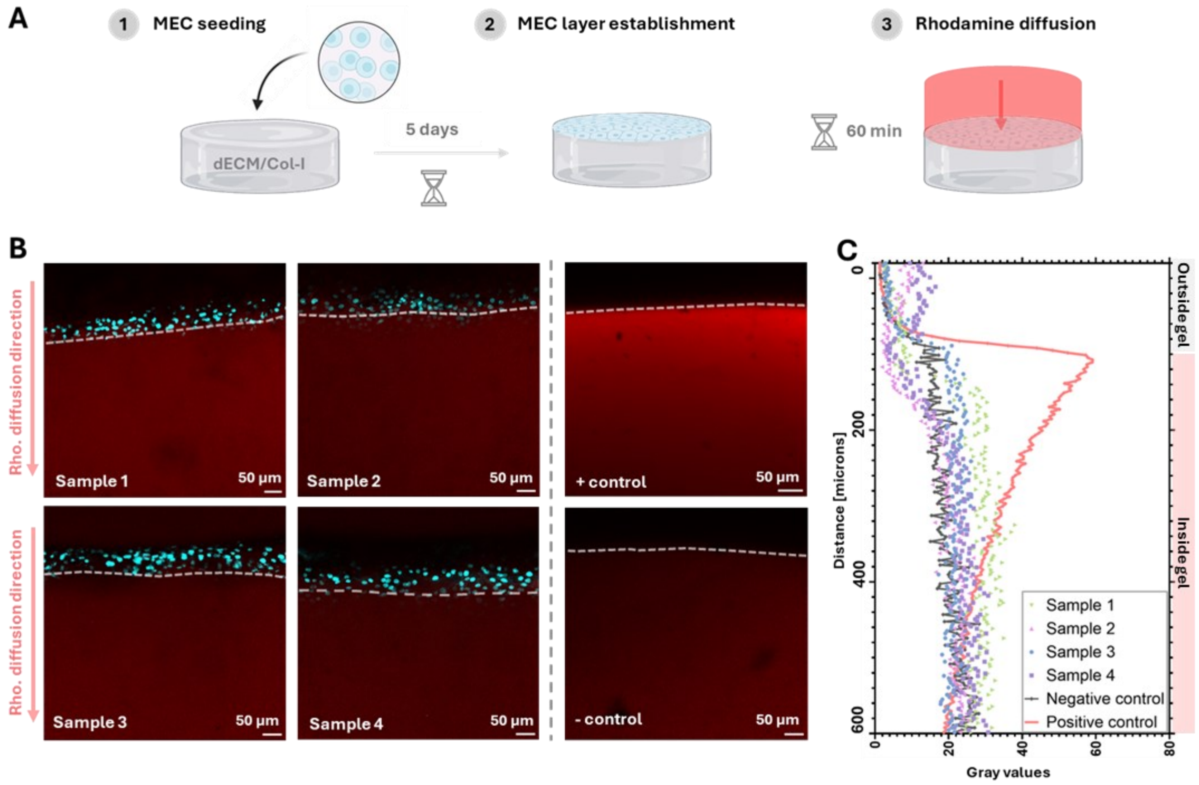

Figure S27: Epithelial barrier assessment on dECM/Collagen-I hydrogels by rhodamine diffusion

A) Experimental set up: (1) Cells were seeded onto dECM/Collagen-I hydrogels and (2) cultured for 5 days to allow the formation of a confluent epithelial layer. (3) A droplet of rhodamine (10kDA) solution was applied onto the epithelial layer and after 60 minutes diffusion was analyzed. B) Representative fluorescence images show the absence of rhodamine in the hydrogel samples compared to the positive and negative controls. The positive control consists of the hydrogel without the MEC layer but with rhodamine, while the negative control consists of the hydrogel without the MEC layer and without rhodamine, used to assess the autofluorescence of the gel. The dashed white line indicates the boundary of the hydrogel. Cells were counterstained with DAPI. Scale bars = 50 µm. C) Quantitative diffusion profiles for each sample, with positive and negative controls shown for reference. Y-axis: distance in microns from the top to the bottom of the acquired image; X-axis: gray values representing rhodamine intensity at the indicated distance.

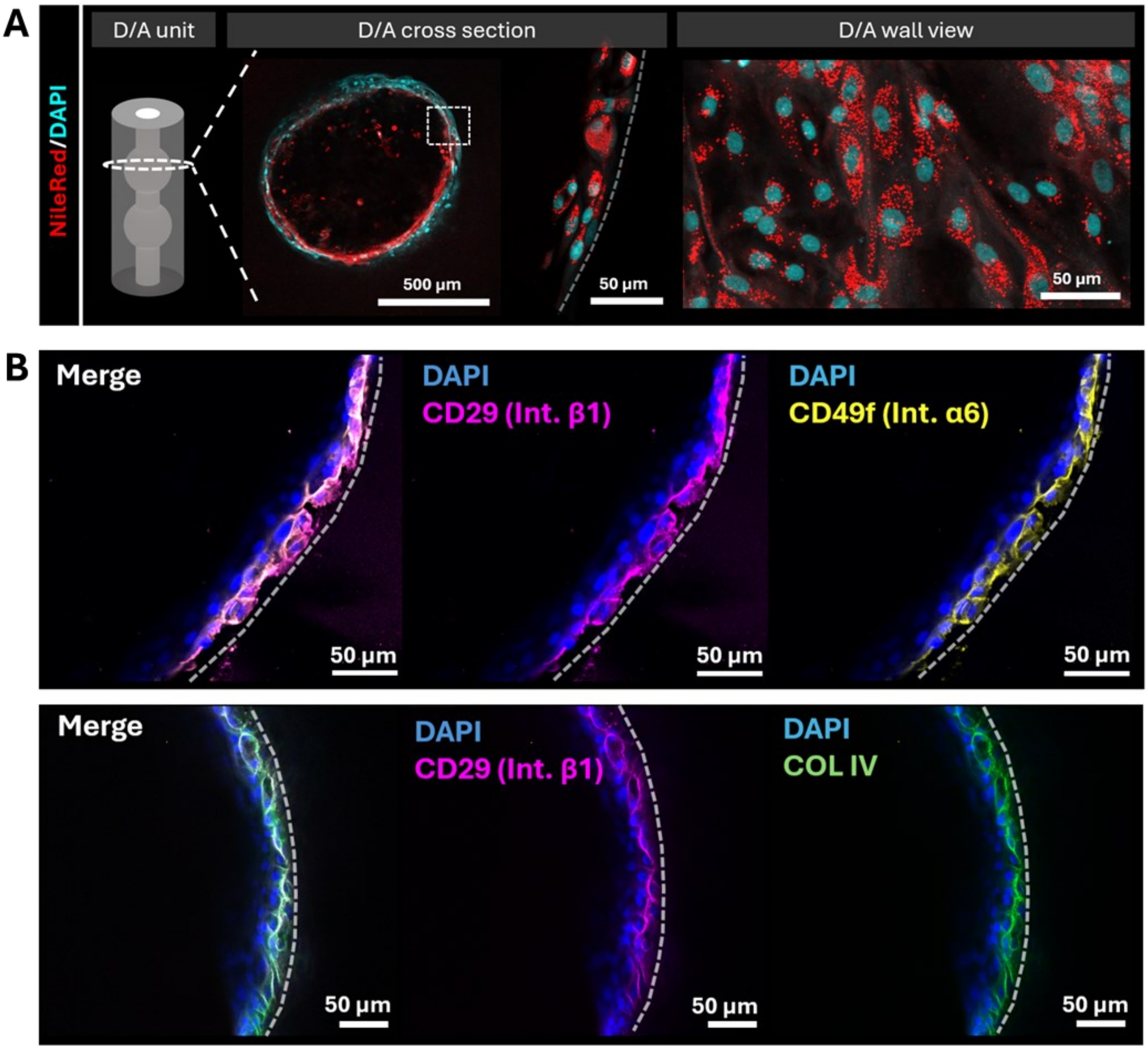

Figure S28: Images of Lipid Accumulation, and Localization of Integrins and Collagen IV in D/A Units

A) Cross-sectional views and high magnification views of D/A unit stained with Nile Red (red, marking neutral lipids) and DAPI (cyan, marking nuclei). The images demonstrate the presence of lipid droplets localized within the epithelial lining of the D/A units. B) Split channels and merged images for D/A units stained for CD29 (pink), CD49f (yellow), Col-IV (green) and DAPI (blue) at day 7 of culture. Scale bars = 50 µm.

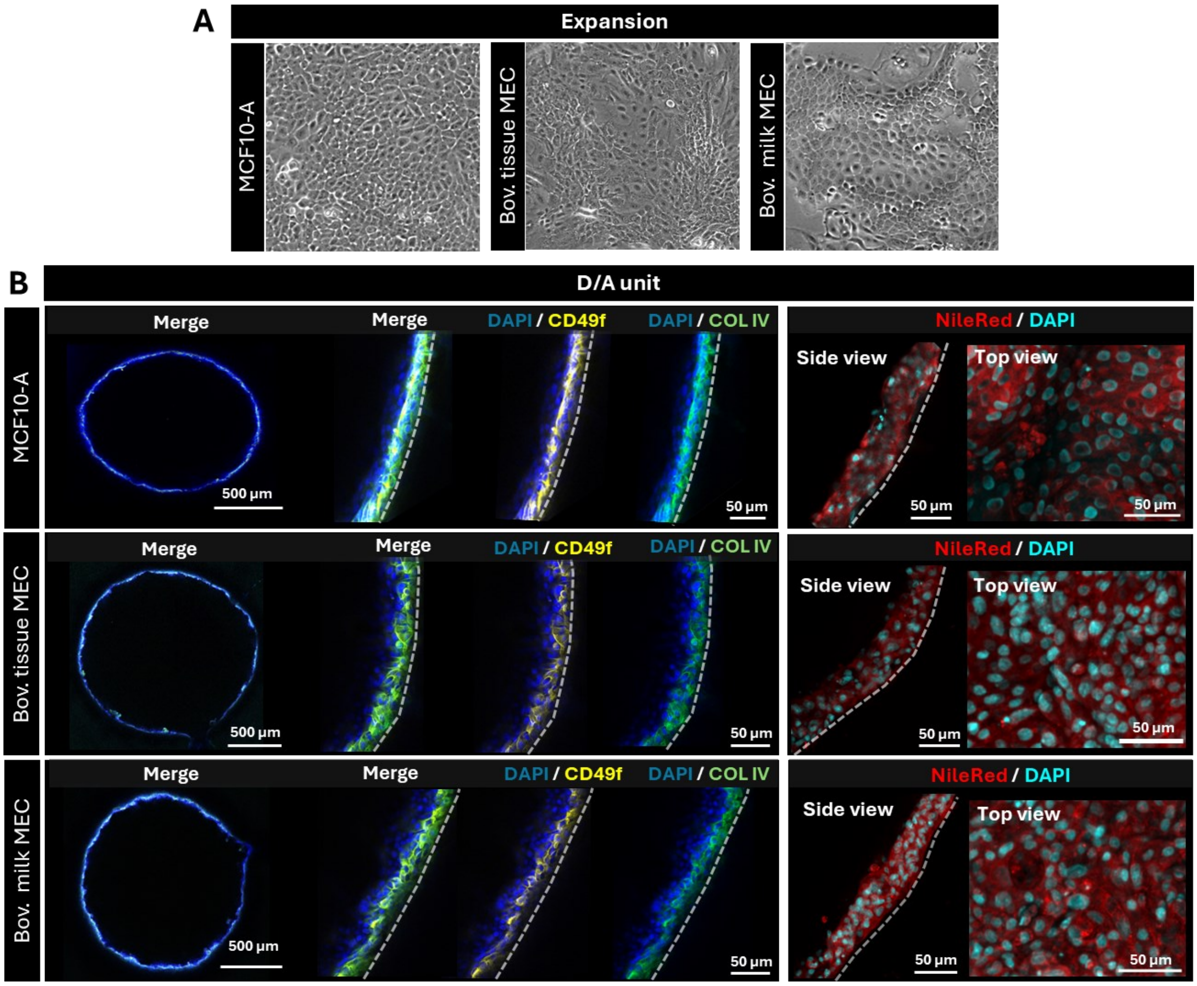

Figure S29: Demonstrating the versatility of D/A units with the MCF10-A breast cell line and bovine-derived MECs.

A) Brightfield images of MCF10-A cells, bovine lactating tissue-derived mammary epithelial cells and bovine milk-derived mammary epithelial cells during 2D expansion used to see the ductal alveolar (D/A) units.
B) Immunofluorescent imaging of the D/A units seeded with MCF10-A, bovine tissue-derived MECs and bovine milk-derived MECs at day 7 of culture. Cell layer was stained for DAPI (blue), CD49f (yellow), Col IV (green) and Nile Red (red).

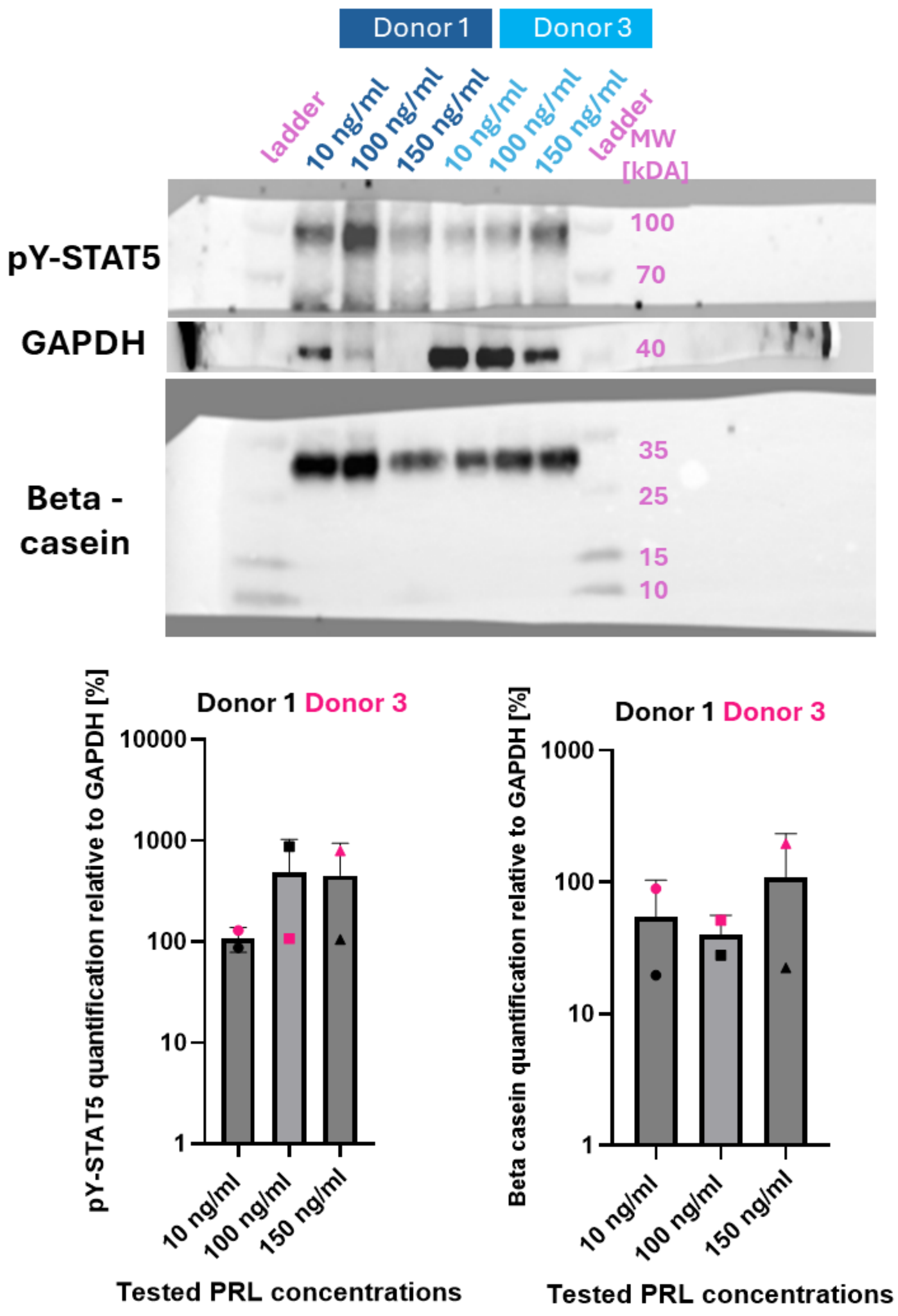

Figure S30: Western Blot analysis of pY-STAT5 and beta casein expression at different PRL concentrations from cells cultured in D/A units.

Western blot for beta casein and pY-STAT5 of milk MEC cultured on our 3D scaffolds with varying PRL concentrations (left) and corresponding quantification (right). The protein ladder and corresponding molecular weights are indicated in purple. The 50 kDa band was excluded to prevent an unspecific band from appearing at that molecular weight, as indicated in the datasheet. Mean ± SD.

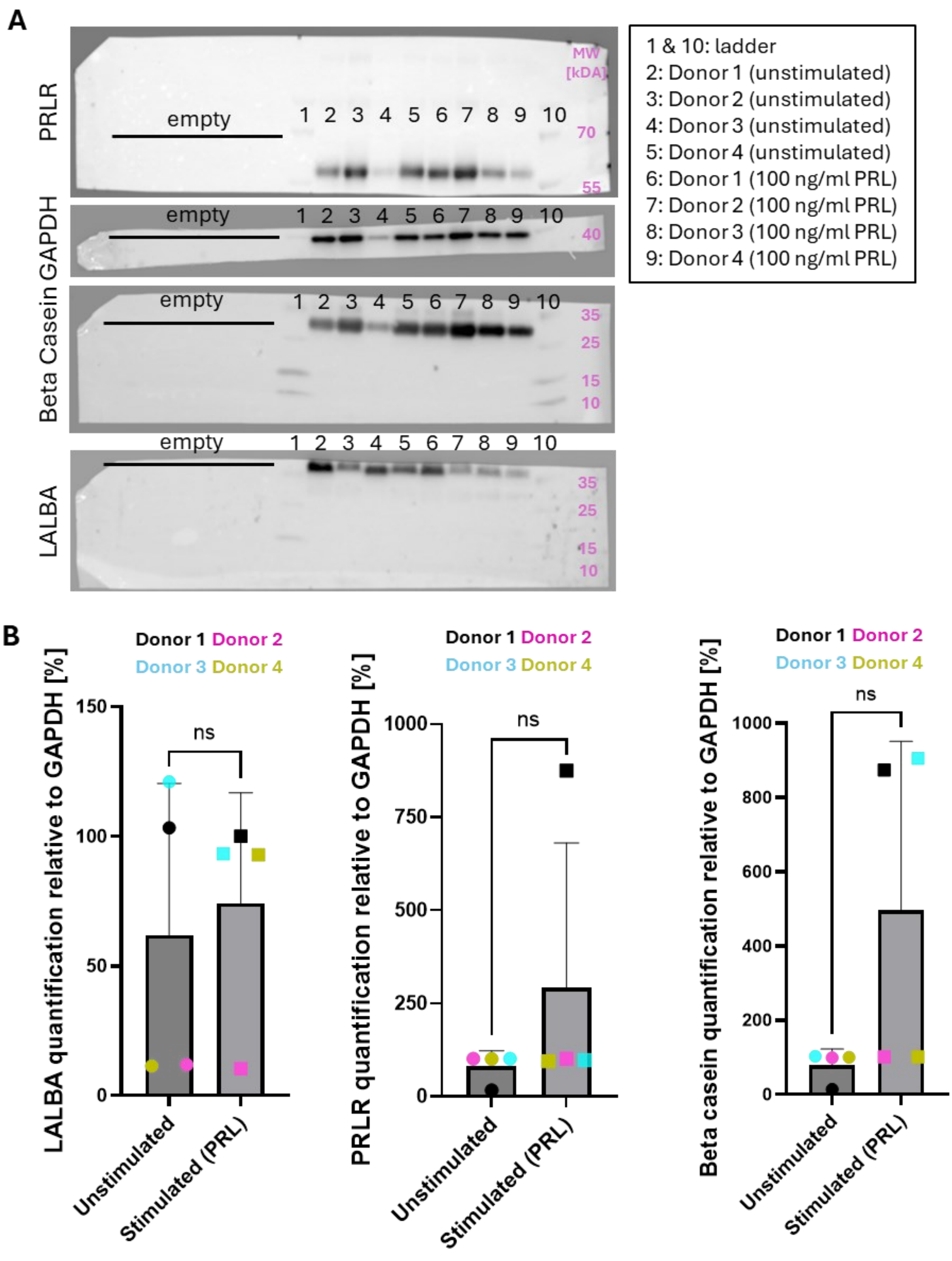

Figure S31: Full Westernblot from main Figure 5.

A) Full Westernblot from main figure 5. Numbers are indicating the lanes, loaded samples are described in the black box. Molecular weight of the used ladder is indicated in purple. B) Westernblot quantification relative to GAPDH from 4 donors from mammary tissue constructs (D/A units) simulated with prolactin (100 ng/ml) vs. unstimulated. Mean ± SD, $p<0.05$.

Sample	Maternal age [years]	BMI	Infant age [weeks]	Parity	Cells / ml
Donor 1	36	23.1	35	2	345K
Donor 2	27	31.7	24	1	734K
Donor 3	35	28.7	16	1	383K
Donor 4	31	21.0	21	1	642 K

Table S1. Demographic information of the participating milk donors.

Authors	Cell type	Species	Culture type	Base med.	FBS	P/S	Insulin	FGF2	FGF10	EGF	HC	Rocki	B27	ITS	Prol.	Oxy.	Prog.	Estr.
Yuan et al. 2023 (57)	Primary MECs	Mouse	3D	DMEM F12 (1:1)	0.50%	1%		20 ng/mL	10 ng/mL			10 µM	10 ng/mL		1 ug/mL		60 nM	
Yuan et al. 2023 (57)	Primary MECs	Mouse	3D	DMEM F12 (1:1)	2%	1%	20 ug/mL			10 ng/mL	1 ug/mL	10 µM	10 ng/mL		1 ug/mL	10 pg/mL		
Sumbal et al. 2020 (58)	Primary MECs	Mouse	3D	DMEM F12 (1:1)	5%	1%					1 µg/mL			1×	1 µg/mL	40 µg/mL		
Jin et al. 2017 (35)	Primary MECs	Human	2D	DMEM/F12 (1:1)	5%				0.4 µg/mL	5 µg/mL	10 ng/mL		24 µg/mL	10 µM				20 nM

Table S2: Lactation media compositions reported in the literature.

Antigen	Fluorochrome	Dilution	Clone	Host / isotype	Reactivity	Company	Cat. Number
CD90	BV510	1:40	5E10	Mouse IgG1, k	Human	Biolegend	328125
CD45	BV785	1:80	HI131	Mouse IgG1, k	Human	Biolegend	304008
CD31	PE	1:640	WM59	Mouse IgG1, k	Human	Biolegend	303106
CD49f	APC	1:160	GoH3	Rat IgG2a, k	Human	Biolegend	313615
EPCAM	FITC	1:160	9C4	Mouse IgG2b, k	Human	Biolegend	324203

Table S3. List of antibodies used for Flow Cytometry.

Antigen	Dilution	Host	Reactivity	Company	Cat. Number
Casein	1:100	Mouse	Human	Abcam	ab47972
Zonulin1	1:100	Rabbit	Human, MS, Rat, Dog, GP	Invitrogen	617300
P63	1:100	Mouse	Human	Abcam	ab735
CK8	1:50	Rabbit	Human	Invitrogen	PA5118024
CK8 TROMA 1	1:50	Rat	Human, MS	Merck	MABT329
CK14	1:100	Rabbit	Human, MS, Rat	Biolegend	905304
EPCAM	1:200	Rabbit	Human, MS, Rat	BIOSSUSA	bs1513R
CD49f	1:100	Rat	Human, Ms	Invitrogen	14-0495-82
Milk Fat Globule	1:100	Mouse	Human	Neo Biotech.	4240-MSM1-P0
Col-III	1:100	Rabbit	Human	Rockland	6000401105S
Col - I	1:100	Mouse	Human	Abcam	ab6308
FOXA1	1:100	Rabbit	Human, Ms	Proteintech	20411-1-AP
SMA	1:100	Mouse	Human, Ms, Rat	Invitrogen	14-9760-82
CD29	1:100	Mouse	Human	Invitrogen	14-0299-82
Col-IV	1:100	Mouse	Human	Invitrogen	14-9871-82

Table S4. List of primary antibodies used for immunostaining.

Secondary Antibody	Host	Dilution	Isotype	Company	Cat. Number
Anti Mouse AF 488	Goat	1:500	IgG (H+L)	Invitrogen	A11001
Anti Rat AF 488	Goat	1:500	IgG (H+L)	Invitrogen	A11006
Anti Rabbit AF 647	Goat	1:500	IgG (H+L)	Invitrogen	A21244
Anti mouse AF 568	Goat	1:500	IgG (H+L)	Invitrogen	A11004
Anti mouse AF 568	Goat	1:500	IgG1 (y1)	Invitrogen	A21124

Table S5. List of secondary antibodies used for immunostaining.

Antigen	Dilution	Clone	Host / isotype	Reactivity	Company	Cat. Number
PRLR	1:1000	2E10G5	Mouse / IgG1	Human	Proteintech	67292
PRL	1:500	C13	Mouse	Human	Invitrogen	MA543711
LALBA	1:1000	Polyclonal	Rabbit	Human	Invitrogen	PA5-56274
Casein	1:500	F20.14	Mouse	Human	Abcam	ab47972
GAPDH	1:10000	Polyclonal	Rabbit / IgG	Human	Invitrogen	PA1-987
FOXA1	1:1000	Polyclonal	Rabbit / IgG	Human, Ms	Proteintech	20411-1-AP
pSTAT5	1:1000	5F6.F1	Mouse / IgG1	Human, Ms, Rat	Rockland	200-301-A45S

Table S6. List of antibodies used for Western Blot.

Gene	Annealing	Oligonucleotide primer sequence
PRLR	FW	5'-TTA CCA CAG GGA AGG AGA GAC A-3'
	REV	5'-GGT GTA CTG CTT GCC AAA GTG-3'
STAT5A	FW	5'-GCC GGT TTG AGT GAG GGT TT-3'
	REV	5'-GGA AAC GTG GGA ACA GCA TC-3'
ELF5	FW	5'-ATT TCC ACT GCA CGC TGG TG-3
	REV	5'-TGT GTG TCA CCG AGT CCA AC-3'
CSN2	FW	5'-CCT CTG AGA CTG ATA GTA TTT-3'
	REV	5'-TGG ATG CTG GAG TGA ACT TTA-3'
GATA3	FW	5'-TGTATGAGCTACCAGCGTGC-3'
	REV	5’-AACACAGAACACGACAGGGC-3'

Table S7. List of RT-qPCR primers.

Supplementary methods

Cell culture of MCF10-A, bovine tissue derived MECs and bovine mil-derived MECs. MCF10-A cells were cultured using established protocols (68).
Bovine tissue-derived and milk-derived MECs were cultured in DMEM/F12 with, 10% FBS and 1% Pen/strep (with media changes every two days) at 37°C under 21% O2. Cells were passaged at 90% confluency.

Lipid droplet staining with Nile Red. Nile Red (catalog no. 19123, Sigma) was dissolved in DMSO to prepare a 10 mM stock solution (3.18 mg/mL). For staining, the stock was diluted in PBS to a final concentration of 10 μM, and Hoechst was added at a 1:100 dilution. Constructs were washed with PBS for 10 minutes, incubated with the staining solution for 15 minutes, washed twice with PBS, and then imaged using an Olympus FV4000 confocal microscope.

Rhodamine diffusion experiments. 10 kDa Rhodamine B isothiocyanate–Dextran (R8881-100MG, Sigma) was dissolved in PBS to prepare a 10 mg/mL stock solution. One microliter of the stock was added to 100 μL of cell culture medium. A droplet of the resulting solution was then applied to epithelial cell layers on 3D-printed dECM/Col-I disks and incubated for 1 hour. Following incubation, the droplet was aspirated, and diffusion was imaged. Diffusion was analyzed using the Surface Plot function in Fiji/ImageJ.

Flow cytometry for Binucleation experiments. Cells were grown to 90% confluency and harvested. They were incubated at RT with CD49f and EPCAM antibodies for 15 minutes, fixed with 4% PFA for 10 minutes, and then stained with DAPI (D9542, Sigma) for 15 minutes to assess ploidy (see Table S3 for the complete antibody list). For EdU staining, we used the Click-iT Plus EdU Alexa Fluor 488 Flow Cytometry Assay kit (C10632, Invitrogen) according to the manufacturer's protocol, followed by a 15-minute DAPI stain prior to flow cytometric analysis. Flow cytometry was performed on a Cytek Aurora Spectral Analyzer, and data was analyzed with FlowJo™ v10.

Cell encapsulation in dECM and Matrigel. Cells were harvested and resuspended in either Matrigel (11573620, Corning) or decellularized extracellular matrix (dECM) to form 30 μL domes containing 10,000 cells each. Human tissue was decellularized using our established protocol for bovine tissue. Prior to encapsulation, the rheological properties of both human and bovine dECM were calibrated to match those of Matrigel. A 50 μL droplet of the cell-matrix suspension was then dispensed into the center of each well of a 24-well suspension culture plate and allowed to solidify at 37 °C for 20 minutes. Finally, 500 μL of culture medium was added to each well, with media changes performed daily.

REFERENCES AND NOTES

1. S. O. Mohamed Ahmed, R. I. Ashgar, A. A. Mohammed Abdelgader, H. I. Abdalla Hamid, D. M. Mathkor, M. A. Abdelsadig Ali, S. A. Mousa Mohmed, S. J. Ali Farg, R. A. Mohamed Ebrahim, M. Mustafa Gabir Tia, W. A. Humza Bashir, Z. H. Ali Hazazi, Exclusive breastfeeding: Impact on infant health. *Clin. Nutr. Open Sci.* **51**, 44–51 (2023).

2. S. Y. Kim, D. Y. Yi, Components of human breast milk: From macronutrient to microbiome and microRNA. *Clin. Exp. Pediatr.* **63**, 301–309 (2020).

3. X. He, M. Parenti, T. Grip, M. Domellöf, B. Lönnerdal, O. Hernell, N. Timby, C. M. Slupsky, Metabolic phenotype of breast-fed infants, and infants fed standard formula or bovine MFGM supplemented formula: A randomized controlled trial. *Sci. Rep.* **9**, 339 (2019).

4. N. V. Bhattacharjee, L. E. Schaeffer, S. I. Hay, Local Burden Of Disease Exclusive Breastfeeding Collaborators, Mapping inequalities in exclusive breastfeeding in low- and middle-income countries, 2000–2018. *Nat. Hum. Behav.* **5**, 1027–1045 (2021).

5. T. McClintock, C. Fiddes, S. Harris, N. Embleton, L. Lin, F. H. Bloomfield, M. Muelbert, Donor human milk versus infant formula for low-risk infants: A systematic review. *Pediatr. Res.* **97**, 81–91 (2025).

6. C. Gale, K. M. Logan, S. Santhakumaran, J. R. Parkinson, M. J. Hyde, N. Modi, Effect of breastfeeding compared with formula feeding on infant body composition: A systematic review and meta-analysis123. *Am. J. Clin. Nutr.* **95**, 656–669 (2012).

7. R. Relaksana, A. Akbar, E. D. Sihaloho, D. Ferdian, A. Y. Siregar, The financial need of feeding infants for the first six months of life in West Java Province of Indonesia and the implications of socioeconomic and mental health factors. *Int. Breastfeed. J.* **18**, 26 (2023).

8. J. Muschler, C. H. Streuli, Cell–matrix interactions in mammary gland development and breast cancer. *Cold Spring Harb. Perspect. Biol.* **2**, a003202 (2010).

9. C. M. Nelson, M. J. Bissell, Of extracellular matrix, scaffolds, and signaling: Tissue architecture regulates development, homeostasis, and cancer. *Annu. Rev. Cell Dev. Biol.* **22**, 287–309 (2006).

10. A. Manninen, Epithelial polarity – Generating and integrating signals from the ECM with integrins. *Exp. Cell Res.* **334**, 337–349 (2015).

11. M. J. Bissell, H. G. Hall, G. Parry, How does the extracellular matrix direct gene expression? *J. Theor. Biol.* **99**, 31–68 (1982).

12. C. M. Ghajar, M. J. Bissell, Extracellular matrix control of mammary gland morphogenesis and tumorigenesis: Insights from imaging. *Histochem. Cell Biol.* **130**, 1105–1118 (2008).

13. J. Muschler, A. Lochter, C. D. Roskelley, P. Yurchenco, M. J. Bissell, Division of labor among the α6β4 integrin, β1 integrins, and an E3 laminin receptor to signal morphogenesis and β-casein expression in mammary epithelial cells. *Mol. Biol. Cell* **10**, 2817–2828 (1999).

14. N. Gjorevski, M. Nikolaev, T. E. Brown, O. Mitrofanova, N. Brandenberg, F. W. DelRio, F. M. Yavitt, P. Liberali, K. S. Anseth, M. P. Lutolf, Tissue geometry drives deterministic organoid patterning. *Science* **375**, eaaw9021 (2022).

15. J. A. Reid, X.-L. Palmer, P. A. Mollica, N. Northam, P. C. Sachs, R. D. Bruno, A 3D bioprinter platform for mechanistic analysis of tumoroids and chimeric mammary organoids. *Sci. Rep.* **9**, 7466 (2019).

16. G. Dontu, T. A. Ince, Of mice and women: A comparative tissue biology perspective of breast stem cells and differentiation. *J. Mammary Gland Biol. Neoplasia* **20**, 51–62 (2015).

17. S. McNally, T. Stein, Overview of mammary gland development: A comparison of mouse and human. *Methods Mol. Biol.* **1501**, 1–17 (2017).

18. R. Doerfler, S. Yerneni, A. Newby, N. Chaudhary, A. Shu, K. Fein, J. Hofstatter Azambuja, K. A. Whitehead, Characterization and comparison of human and mouse milk cells. *PLOS ONE* **19**, e0297821 (2024).

19. S. Görs, M. Kucia, M. Langhammer, P. Junghans, C. C. Metges, Technical note: Milk composition in mice—Methodological aspects and effects of mouse strain and lactation day. *J. Dairy Sci.* **92**, 632–637 (2009).

20. B. Blanco-Fernandez, S. Rey-Vinolas, G. Bağcı, G. Rubi-Sans, J. Otero, D. Navajas, S. Perez-Amodio, E. Engel, Bioprinting decellularized breast tissue for the development of three-dimensional breast cancer models. *ACS Appl. Mater. Interfaces* **14**, 29467–29482 (2022).

21. P. Barros da Silva, M. Coelho, S. J. Bidarra, S. C. Neves, C. C. Barrias, Reshaping in vitro models of breast tissue: Integration of stromal and parenchymal compartments in 3D printed hydrogels. *Front. Bioeng. Biotechnol.* **8**, 494 (2020).

22. P. A. Mollica, E. N. Booth-Creech, J. A. Reid, M. Zamponi, S. M. Sullivan, X.-L. Palmer, P. C. Sachs, R. D. Bruno, 3D bioprinted mammary organoids and tumoroids in human mammary derived ECM hydrogels. *Acta Biomater.* **95**, 201–213 (2019).

23. P. Chansoria, D. Rütsche, A. Wang, H. Liu, D. D'Angella, R. Rizzo, A. Hasenauer, P. Weber, W. Qiu, N. B. M. Ibrahim, N. Korshunova, X.-H. Qin, M. Zenobi-Wong, Synergizing algorithmic design, photoclick chemistry and multi-material volumetric printing for accelerating complex shape engineering. *Adv. Sci.* **10**, e2300912 (2023).

24. R. Rizzo, D. Ruetsche, H. Liu, M. Zenobi-Wong, Optimized photoclick (bio)resins for fast volumetric bioprinting. *Adv. Mater.* **33**, e2102900 (2021).

25. H. Kim, B. Kang, X. Cui, S.-H. Lee, K. Lee, D.-W. Cho, W. Hwang, T. B. F. Woodfield, K. S. Lim, J. Jang, Light-activated decellularized extracellular matrix-based bioinks for volumetric tissue analogs at the centimeter scale. *Adv. Funct. Mater.* **31**, 2011252 (2021).

26. L. Lian, M. Xie, Z. Luo, Z. Zhang, S. Maharjan, X. Mu, C. E. Garciamendez-Mijares, X. Kuang, J. K. Sahoo, G. Tang, G. Li, D. Wang, J. Guo, F. Z. González, V. A. M. Rivera, L. Cai, X. Mei, D. L. Kaplan, Y. S. Zhang, Rapid volumetric bioprinting of decellularized extracellular matrix bioinks. *Adv. Mat.* **36**, 2304846 (2024).

27. J. D. Hunter, T. D. Johnson, R. L. Braden, K. L. Christman, "Injectable ECM scaffolds for cardiac repair," in *Cardiac Tissue Engineering: Methods and Protocols*, Methods in Molecular Biology, K. L. K. Coulombe, L. D. Black III, Eds. (Springer, 2022), pp. 255–268.

28. X. Zhang, X. Chen, H. Hong, R. Hu, J. Liu, C. Liu, Decellularized extracellular matrix scaffolds: Recent trends and emerging strategies in tissue engineering. *Bioact. Mater.* **10**, 15–31 (2021).

29. G. G. Giobbe, C. Crowley, C. Luni, S. Campinoti, M. Khedr, K. Kretzschmar, M. M. De Santis, E. Zambaiti, F. Michielin, L. Meran, Q. Hu, G. van Son, L. Urbani, A. Manfredi, M. Giomo, S. Eaton, D. Cacchiarelli, V. S. W. Li, H. Clevers, P. Bonfanti, N. Elvassore, P. De Coppi, Extracellular matrix hydrogel derived from decellularized tissues enables endodermal organoid culture. *Nat. Commun.* **10**, 5658 (2019).

30. J. F. Martin Carli, G. D. Trahan, K. L. Jones, N. Hirsch, K. P. Rolloff, E. Z. Dunn, J. E. Friedman, L. A. Barbour, T. L. Hernandez, P. S. MacLean, J. Monks, J. L. McManaman, M. C. Rudolph, Single cell RNA sequencing of human milk-derived cells reveals sub-populations of mammary epithelial cells with molecular signatures of progenitor and mature states: A novel, non-invasive framework for investigating human lactation physiology. *J. Mammary Gland Biol. Neoplasia* **25**, 367–387 (2020).

31. J. P. Gleeson, N. Chaudhary, K. C. Fein, R. Doerfler, P. Hredzak-Showalter, K. A. Whitehead, Profiling of mature-stage human breast milk cells identifies six unique lactocyte subpopulations. *Sci. Adv.* **8**, eabm6865 (2022).

32. A.-J. Twigger, L. K. Engelbrecht, K. Bach, I. Schultz-Pernice, S. Pensa, J. Stenning, S. Petricca, C. H. Scheel, W. T. Khaled, Transcriptional changes in the mammary gland during lactation revealed by single cell sequencing of cells from human milk. *Nat. Commun.* **13**, 562 (2022).

33. F. Hassiotou, A. Beltran, E. Chetwynd, A. M. Stuebe, A.-J. Twigger, P. Metzger, N. Trengove, C. T. Lai, L. Filgueira, P. Blancafort, P. E. Hartmann, Breastmilk is a novel source of stem cells with multilineage differentiation potential. *Stem Cells* **30**, 2164–2174 (2012).

34. R. Ragazzini, S. Boeing, L. Zanieri, M. Green, G. D'Agostino, K. Bartolovic, A. Agua-Doce, M. Greco, S. A. Watson, A. Batsivari, L. Ariza-McNaughton, A. Gjinovci, D. Scoville, A. Nam, A. C. Hayday, D. Bonnet, P. Bonfanti, Defining the identity and the niches of epithelial stem cells with highly pleiotropic multilineage potency in the human thymus. *Dev. Cell* **58**, 2428–2446.e9 (2023).

35. L. Jin, Y. Qu, L. J. Gomez, S. Chung, B. Han, B. Gao, Y. Yue, Y. Gong, X. Liu, F. Amersi, C. Dang, A. E. Giuliano, X. Cui, Characterization of primary human mammary epithelial cells isolated and propagated by conditional reprogrammed cell culture. *Oncotarget* **9**, 11503–11514 (2018).

36. G. K. Gray, C. M.-C. Li, J. M. Rosenbluth, L. M. Selfors, N. Girnius, J.-R. Lin, R. C. J. Schackmann, W. L. Goh, K. Moore, H. K. Shapiro, S. Mei, K. D'Andrea, K. L. Nathanson, P. K. Sorger, S. Santagata, A. Regev, J. E. Garber, D. A. Dillon, J. S. Brugge, A human breast atlas integrating single-cell proteomics and transcriptomics. *Dev. Cell* **57**, 1400–1420.e7 (2022).

37. Ö. Karayazi Atıcı, N. Govindrajan, I. Lopetegui-González, C. S. Shemanko, Prolactin: A hormone with diverse functions from mammary gland development to cancer metastasis. *Semin. Cell Dev. Biol.* **114**, 159–170 (2021).

38. N. Ben-Jonathan, J. L. Mershon, D. L. Allen, R. W. Steinmetz, Extrapituitary prolactin: Distribution, regulation, functions, and clinical aspects. *Endocr. Rev.* **17**, 639–669 (1996).

39. T. Iwasaka, S. Umemura, K. Kakimoto, H. Koizumi, Y. R. Osamura, Expression of prolactin mRNA in rat mammary gland during pregnancy and lactation. *J. Histochem. Cytochem.* **48**, 389–395 (2000).

40. S. K. Nyquist, P. Gao, T. K. J. Haining, M. R. Retchin, Y. Golan, R. S. Drake, K. Kolb, B. E. Mead, N. Ahituv, M. E. Martinez, A. K. Shalek, B. Berger, B. A. Goods, Cellular and transcriptional diversity over the course of human lactation. *Proc. Natl. Acad. Sci. U.S.A.* **119**, e2121720119 (2022).

41. G. M. Bernardo, K. L. Lozada, J. D. Miedler, G. Harburg, S. C. Hewitt, J. D. Mosley, A. K. Godwin, K. S. Korach, J. E. Visvader, K. H. Kaestner, F. W. Abdul-Karim, M. M. Montano, R. A. Keri, FOXA1 is an essential determinant of ERα expression and mammary ductal morphogenesis. *Development* **137**, 2045–2054 (2010).

42. N. Forster, S. V. Saladi, M. van Bragt, M. E. Sfondouris, F. E. Jones, Z. Li, L. W. Ellisen, Basal cell signaling by p63 controls luminal progenitor function and lactation via NRG1. *Dev. Cell* **28**, 147–160 (2014).

43. A. C. Rios, N. Y. Fu, P. R. Jamieson, B. Pal, L. Whitehead, K. R. Nicholas, G. J. Lindeman, J. E. Visvader, Essential role for a novel population of binucleated mammary epithelial cells in lactation. *Nat. Commun.* **7**, 11400 (2016).

44. K. Hughes, Comparative mammary gland postnatal development and tumourigenesis in the sheep, cow, cat and rabbit: Exploring the menagerie. *Semin. Cell Dev. Biol.* **114**, 186–195 (2021).

45. C. R. Keller, K. F. Ruud, S. R. Martinez, W. Li, Identification of the collagen types essential for mammalian breast acinar structures. *Gels* **8**, 837 (2022).

46. X. Shao, I. N. Taha, K. R. Clauser, Y. Gao, A. Naba, MatrisomeDB: The ECM-protein knowledge database. *Nucleic Acids Res.* **48**, D1136 (2019).

47. M.-S. Kim, S. M. Pinto, D. Getnet, R. S. Nirujogi, S. S. Manda, R. Chaerkady, A. K. Madugundu, D. S. Kelkar, R. Isserlin, S. Jain, J. K. Thomas, B. Muthusamy, P. Leal-Rojas, P. Kumar, N. A. Sahasrabuddhe, L. Balakrishnan, J. Advani, B. George, S. Renuse, L. D. N. Selvan, A. H. Patil, V. Nanjappa, A. Radhakrishnan, S. Prasad, T. Subbannayya, R. Raju, M. Kumar, S. K. Sreenivasamurthy, A. Marimuthu, G. J. Sathe, S. Chavan, K. K. Datta, Y. Subbannayya, A. Sahu, S. D. Yelamanchi, S. Jayaram, P. Rajagopalan, J. Sharma, K. R. Murthy, N. Syed, R. Goel, A. A. Khan, S. Ahmad, G. Dey, K. Mudgal, A. Chatterjee, T.-C. Huang, J. Zhong, X. Wu, P. G. Shaw, D. Freed, M. S. Zahari, K. K. Mukherjee, S. Shankar, A. Mahadevan, H. Lam, C. J. Mitchell, S. K. Shankar, P. Satishchandra, J. T. Schroeder, R. Sirdeshmukh, A. Maitra, S. D. Leach, C. G. Drake, M. K. Halushka, T. S. K. Prasad, R. H.

Hruban, C. L. Kerr, G. D. Bader, C. A. Iacobuzio-Donahue, H. Gowda, A. Pandey, A draft map of the human proteome. *Nature* **509**, 575–581 (2014).

48. K. Elkhoury, J. Zuazola, S. Vijayavenkataraman, Bioprinting the future using light: A review on photocrosslinking reactions, photoreactive groups, and photoinitiators. *SLAS Technol.* **28**, 142–151 (2023).

49. K. Hughes, C. J. Watson, The spectrum of STAT functions in mammary gland development. *JAKSTAT* **1**, 151–158 (2012).

50. M. Miyano, M. A. LaBarge, ELF5: A molecular clock for breast aging and cancer susceptibility. *Cancers* **16**, 431 (2024).

51. H. Kouros-Mehr, J. Kim, S. K. Bechis, Z. Werb, GATA-3 and the regulation of the mammary luminal cell fate. *Curr. Opin. Cell Biol.* **20**, 164–170 (2008).

52. P. A. Abramicheva, O. V. Smirnova, Prolactin receptor isoforms as the basis of tissue-specific action of prolactin in the norm and pathology. *Biochemistry* **84**, 329–345 (2019).

53. K. E. Boonekamp, K. Kretzschmar, D. J. Wiener, P. Asra, S. Derakhshan, J. Puschhof, C. López-Iglesias, P. J. Peters, O. Basak, H. Clevers, Long-term expansion and differentiation of adult murine epidermal stem cells in 3D organoid cultures. *Proc. Natl. Acad. Sci. U.S.A.* **116**, 14630–14638 (2019).

54. M. Nikolaev, O. Mitrofanova, N. Broguiere, S. Geraldo, D. Dutta, Y. Tabata, B. Elci, N. Brandenberg, I. Kolotuev, N. Gjorevski, H. Clevers, M. P. Lutolf, Homeostatic mini-intestines through scaffold-guided organoid morphogenesis. *Nature* **585**, 574–578 (2020).

55. J. M. Rosenbluth, R. C. J. Schackmann, G. K. Gray, L. M. Selfors, C. M.-C. Li, M. Boedicker, H. J. Kuiken, A. Richardson, J. Brock, J. Garber, D. Dillon, N. Sachs, H. Clevers, J. S. Brugge, Organoid cultures from normal and cancer-prone human breast tissues preserve complex epithelial lineages. *Nat. Commun.* **11**, 1711 (2020).

56. L. Yuan, S. Xie, H. Bai, X. Liu, P. Cai, J. Lu, C. Wang, Z. Lin, S. Li, Y. Guo, S. Cai, Reconstruction of dynamic mammary mini gland in vitro for normal physiology and oncogenesis. *Nat. Methods,* **20**, 2021–2033 (2023).

57. J. Sumbal, A. Chiche, E. Charifou, Z. Koledova, H. Li, Primary mammary organoid model of lactation and involution. *Front. Cell Dev. Biol.* **8**, 68 (2020).

58. T. Jardé, B. Lloyd-Lewis, M. Thomas, H. Kendrick, L. Melchor, L. Bougaret, P. D. Watson, K. Ewan, M. J. Smalley, T. C. Dale, Wnt and neuregulin1/ErbB signalling extends 3D culture of hormone responsive mammary organoids. *Nat. Commun.* **7**, 13207 (2016).

59. K. M. Mak, R. Mei, Basement membrane type IV collagen and laminin: An overview of their biology and value as fibrosis biomarkers of liver disease. *Anat. Rec.* **300**, 1371–1390 (2017).

60. J. I. Englund, A. Ritchie, L. Blaas, H. Cojoc, N. Pentinmikko, J. Döhla, S. Iqbal, M. Patarroyo, P. Katajisto, Laminin alpha 5 regulates mammary gland remodeling through luminal cell differentiation and Wnt4-mediated epithelial crosstalk. *Development* **148**, dev199281 (2021).

61. T. A. Stewart, K. Hughes, A. J. Stevenson, N. Marino, A. L. Ju, M. Morehead, F. M. Davis, Mammary mechanobiology – Investigating roles for mechanically activated ion channels in lactation and involution. *J. Cell Sci.* **134**, jcs248849 (2021).

62. I. S. Kinstlinger, G. A. Calderon, M. K. Royse, A. K. Means, B. Grigoryan, J. S. Miller, Perfusion and endothelialization of engineered tissues with patterned vascular networks. *Nat. Protoc.* **16**, 3089–3113 (2021).

63. L. Moroni, J. A. Burdick, C. Highley, S. J. Lee, Y. Morimoto, S. Takeuchi, J. J. Yoo, Biofabrication strategies for 3D in vitro models and regenerative medicine. *Nat. Rev. Mater.* **3**, 21–37 (2018).

64. J. L. Ungerleider, T. D. Johnson, N. Rao, K. L. Christman, Fabrication and characterization of injectable hydrogels derived from decellularized skeletal and cardiac muscle. *Methods* **84**, 53–59 (2015).

65. J. R. Wiśniewski, A. Zougman, N. Nagaraj, M. Mann, Universal sample preparation method for proteome analysis. *Nat. Methods* **6**, 359–362 (2009).

66. F. F. Voigt, D. Kirschenbaum, E. Platonova, S. Pagès, R. A. A. Campbell, R. Kastli, M. Schaettin, L. Egolf, A. van der Bourg, P. Bethge, K. Haenraets, N. Frézel, T. Topilko, P. Perin, D. Hillier, S. Hildebrand, A. Schueth, A. Roebroeck, B. Roska, E. T. Stoeckli, R. Pizzala, N. Renier, H. U. Zeilhofer, T. Karayannis, U. Ziegler, L. Batti, A. Holtmaat, C. Lüscher, A. Aguzzi, F. Helmchen, The mesoSPIM initiative: Open-source light-sheet microscopes for imaging cleared tissue. *Nat. Methods* **16**, 1105–1108 (2019).

67. O. Chaudhuri, J. Cooper-White, P. A. Janmey, D. J. Mooney, V. B. Shenoy, Effects of extracellular matrix viscoelasticity on cellular behaviour. *Nature* **584**, 535–546 (2020).

68. Y. Qu, B. Han, Y. Yu, W. Yao, S. Bose, B. Y. Karlan, A. E. Giuliano, X. Cui, Evaluation of MCF10A as a reliable model for normal human mammary epithelial cells. *PLOS ONE* **10**, e0131285 (2015).
